# Severe hydroxymethylbilane synthase deficiency causes depression-like behavior and mitochondrial dysfunction in a mouse model of homozygous dominant acute intermittent porphyria

**DOI:** 10.1186/s40478-020-00910-z

**Published:** 2020-03-20

**Authors:** Stefanie Berger, Miranda Stattmann, Ana Cicvaric, Francisco J. Monje, Pierluca Coiro, Matej Hotka, Gerda Ricken, Johannes Hainfellner, Susanne Greber-Platzer, Makiko Yasuda, Robert J. Desnick, Daniela D. Pollak

**Affiliations:** 1 Department of Neurophysiology and Neuropharmacology, Center of Physiology and Pharmacology, Medical University of Vienna, Schwarzspanierstrasse, 17, A-1090 Vienna, Austria; 2 Department of Neurology, Division of Neuropathology and Neurochemistry, Medical University of Vienna, Vienna, Austria; 3 Department of Pediatric and Adolescent Medicine, Medical University of Vienna, Vienna, Austria; 4 Icahn School of Medicine at Mount Sinai, New York, NY USA

**Keywords:** Hippocampus, Hydroxymethylbilane synthase, Mitochondria, Mouse behavior, Porphyria

## Abstract

Acute intermittent porphyria (AIP) is an autosomal dominant inborn error of heme biosynthesis due to a pathogenic mutation in the *Hmbs* gene, resulting in half-normal activity of hydroxymethylbilane synthase. Factors that induce hepatic heme biosynthesis induce episodic attacks in heterozygous patients. The clinical presentation of acute attacks involves the signature neurovisceral pain and may include psychiatric symptoms. Here we used a knock-in mouse line that is biallelic for the *Hmbs* c.500G > A (p.R167Q) mutation with ~ 5% of normal hydroxymethylbilane synthase activity to unravel the consequences of severe HMBS deficiency on affective behavior and brain physiology. *Hmbs* knock-in mice (KI mice) model the rare homozygous dominant form of AIP and were used as tool to elucidate the hitherto unknown pathophysiology of the behavioral manifestations of the disease and its neural underpinnings. Extensive behavioral analyses revealed a selective depression-like phenotype in *Hmbs* KI mice; transcriptomic and immunohistochemical analyses demonstrated aberrant myelination. The uncovered compromised mitochondrial function in the hippocampus of knock-in mice and its ensuing neurogenic and neuroplastic deficits lead us to propose a mechanistic role for disrupted mitochondrial energy production in the pathogenesis of the behavioral consequences of severe HMBS deficiency and its neuropathological sequelae in the brain.

## Introduction

Acute intermittent porphyria (AIP), an autosomal dominant inborn error of heme biosynthesis, is due to pathogenic mutations in the hydroxymethylbilane synthase (*Hmbs*) gene which reduce the activity of its encoded enzyme (HMBS or porphobilinogen deaminase) by approximately 50% [1, 2]. AIP heterozygotes are at risk of having life-threatening acute neurovisceral attacks that may be accompanied by psychiatric manifestations [3–7]. These episodic attacks are triggered by endogenous (e.g. hormones) or exogenous (e.g. drugs) precipitating factors that induce the hepatic expression of δ-aminolevulinic acid synthase 1, the first and rate-limiting enzyme of heme biosynthesis [8–11] resulting in the massive accumulation of the putatively neurotoxic porphyrin precursors, δ-aminolevulinic acid (ALA) and porphobilinogen (PBG) in the liver plasma and urine.

Patients with the rare homozygous dominant form of AIP (HD-AIP) carry biallelic mutations in the *Hmbs* gene and have constitutively elevated levels of urinary ALA and PBG due to a ~ 95% decrease in HMBS activity [12–14]. The clinical presentation of HD-AIP is dominated by neurological impairments and psychomotor retardation [13], which are present without exposure to precipitating porphyrinogenic factors.

An AIP mouse model with ~ 30% of normal HMBS activity (designated T1/T2 mice) was generated over 20 years ago and used as valuable tool for the investigation of the pathogenesis of AIP and exploration of novel therapeutic strategies for the disease [15–17]. As the clinical and biochemical phenotype in these animals is only apparent after induction with a porphyrinogenic agent, namely phenobarbital [18] which has strong sedative effects, the possibilities for behavioral examinations in this model have been limited.

Recently, a mouse strain carrying a c.500G > A knock-in (KI) mutation in the *Hmbs* gene (p.Arg167Glu; R167Q) was generated, with KI mice displaying striking clinical reminiscence of HD-AIP patients [13]. KI mice with only ~ 5% residual murine HMBS activity, have inherently augmented plasma and urine levels of ALA and PBG without induction by a porphyrinogenic drug. These mice also have markedly elevated ALA and PBG concentrations in the cerebral spinal fluid and brain and present with aberrant motor development and defective myelination [13].

Here we hypothesized that characterization of this mouse line and associated molecular underpinnings bears the potential to reveal the consequences of severe HMBS deficiency on behavioral traits and to uncover the underlying pathomechanistic principles. To define the molecular signature paralleling the behavioral phenotype of KI mice we conducted transcriptomic analysis using RNAseq with subsequent immunohistochemical corroboration. We then pursued the possibility that a mitochondrial energy deficit could underlie the observed phenotypic and molecular derangements and defined its consequences for adult hippocampal neurogenesis and synaptic plasticity.

## Materials and methods

### Aim, design and setting of the study

The aim of this study was to examine the consequences of severe HMBS deficiency on affective behavior and to examine the underlying pathophysiological principles in a mouse model of homozygous AIP. KI mice harboring a point mutation in the HMBS gene were compared to WT littermate controls in behavioral, molecular and biochemical studies.

### Animals

Heterozygous breeding couples of the R167Q mouse line [13] were generously provided by Makiko Yasuda, MD, PhD, Icahn School of Medicine at Mount Sinai, NY, USA. For all studies, adult homozygous knock-in mutants (KI; p.Arg167Glu; R167Q mice) were compared to sex- and age-matched wildtype (WT) littermate controls.

Animals were single housed under standard conditions in a colony room with a temperature of 22 ± 1 °C, humidity of 50%, CO_2_ controlled to 500-600 ppm, 12:12 h light/dark cycle and food and water available ad libitum unless stated otherwise. The light intensity amounted to 10-20 lx inside the home cages.

For all behavioral, cellular and molecular analyses adult animals (8–12 weeks) were used.

### Behavioral analyses

Behavioral characterization of KI and WT controls consisted of the following tests:

#### Home cage activity

Daily home cage activity patterns were tracked and recorded using the PhenoTyper® system (Noldus, Wageningen, Netherlands). Mice were placed into the PhenoTyper arena (30 × 30 cm) and continuously monitored for 24 h. The EthoVision software (Noldus, Wageningen, Netherlands) was used to determine and quantify relevant behavioral parameters, including total mobility, distance covered and velocity.

#### Sucrose preference test (SPT)

The SPT assessing anhedonic behavior was adapted from a standard protocol [19–21]. Briefly, adult, single -housed mice were water and food-deprived for 18 h before the 2 days habituation period to 2% sucrose (Sigma-Aldrich, St. Louis, USA) solution. During the 3 h testing period, mice were given the choice to drink from either of two identical bottles, one filled with 2% sucrose solution, the other with regular tap water. The weight of the bottles before and after testing was determined and used for the calculation of the percentage of sucrose preference relative to the total liquid consumption.

#### Novelty suppressed feeding (NSF)

The NSF test was conducted as previously described [22, 23]. Briefly, mice were weighed and food deprived 24 h prior to behavioral testing. A single food pellet (fixed to a circular paper) was placed into the center of a brightly lit (800 lx) arena (30 × 50 cm). The latency to feed on the pellet was recorded (15 min max time) for each mouse and used as a parameter for the indication of depression-related anxiety.

#### Light/dark box (LD)

The LD test was conducted to measure anxiety-like behavior according to a standardized protocol [24] using the Activity Monitor® (MedAssociates Inc., St. Albans, VT, USA). Illumination in the light and dark compartment was maintained constant at 200 lx and 5 lx, respectively. At the beginning of the test, animals were placed into the dark box and allowed to freely explore the arena for 10 min. The amount of time an animal spent in the light area and the transition between the zones was recorded.

#### Elevated plus maze (EPM)

Anxiety-like behavior in the EPM was assessed as previously described [24]. Briefly, the plus maze, consisting of two open and two closed arms, was elevated 65 cm above the floor. Lighting in the open arms amounted to 100 lx and in the closed arms to 30 lx. At the onset of the 5 min trial, mice were placed in the center of the maze facing the left open arm. The time the animal spent in the open versus closed arms was recorded by the software Videotrack® (Viewpoint, Champagne au Mont d’Or, France) and used as a parameter for the indication of anxiety-related behavior.

#### Tail suspension test (TST)

In the TST, behavioral despair was evaluated according to a standard protocol [25]. In short, mice were suspended by their tail in a strain gauge position using a commercial cubicle TST system (MedAssociates Inc., St. Albans, VT, USA). A monitoring system recorded the amount of immobile and mobile time for each animal during the 6 min testing period. The duration of immobility was considered to be an indicator of behavioral despair. SSRI responsivity was tested by acute administration (intraperiteoneal (i.p.)) of Escitalopram (Sigma-Aldrich, St. Louis, USA) (10 mg/kg in 0.9% NaCl) 30 min prior to the TST [26].

### Adult hippocampal neurogenesis

Progenitor cell proliferation and survival of newborn cells were determined based upon published 5-bromodeoxyuridine (BrdU) injection protocols [19, 24]. For the evaluation of progenitor cell proliferation mice were administrated 10 μl/g body weight of 50 mg/kg BrdU (Sigma-Aldrich, St. Louis, USA) dissolved in 0.9% NaCl (i.p.) 5 times in 2 h intervals and perfused 24 h after the last injection. Survival and differentiation of newborn cells were tracked by the application of BrdU (50 mg/kg as above) twice per day (8 h intervals) for 3 consecutive days and perfusion 2 weeks after the first injection. Transcardial in-situ perfusions were performed in deeply anesthetized mice (40% Esketaminhydrochlorid (Ketanest®, Pfizer, New York, USA), 20% xylazine (Rompun®, Bayer, Leverkusen, Germany) in 0.9% NaCl) using 4% paraformaldehyde solution (PFA, Sigma-Aldrich, St. Louis, USA) in 0.1 M phosphate-buffered NaCl. Perfused brains were dissected out, kept overnight in 10 ml of 4% PFA and 2 days in 30% sucrose solution at 4 °C after which whole brains were frozen in O.C.T. (Tissue-Tek Fisher Scientific, Hampton, USA) and stored at − 80 °C until further processing.

#### Immuno-fluorescence-histochemistry

Coronal brain sections (30 μm) containing the hippocampus were cut on a cryostat (CM1950, Leica, Wetzlar, Germany) using the Mouse Brain Atlas as a reference [27]. Free-floating brain sections were kept in a cryoprotectant solution (30% glycerol, 30% ethylene glycol and 40% phosphate-buffered NaCl; pH 7.4). Every 10th section of the entire rostro-caudal span of the hippocampus was used for the quantification of newborn cells, their differentiation and survival using a published immunofluorescence-histochemistry procedure [24, 28–31]. For single staining (proliferation paradigm) only mouse anti-BrdU antibody (Bio-Rad AbD Serotec, United Kingdom; 1:300) was used. Double-staining protocols employed rat anti-BrdU antibody (Bio-Rad AbD Serotec, United Kingdom; 1:300) combined with goat anti-DCX (Santa Cruz, USA, 1:250), mouse anti-NeuN (Millipore Merck, Germany, 1:1000) or rabbit anti-GFAP (Sigma-Aldrich, St. Louis, USA, 1:500). Secondary antibodies were 488 donkey anti-goat (Thermofisher Scientific, Waltham, USA, 1:200), 488 goat anti-mouse (Thermofisher Scientific, Waltham, USA, 1:500), 488 goat anti-rabbit (Thermofisher Scientific, Waltham, USA, 1:500) and 594 chicken anti-rat (Thermofisher Scientific, Waltham, USA, 1:500).

Fluorescence microscopy pictures were acquired on a Carl-Zeiss Axiovert-Apotome System (Oberkochen, Germany) using the Axiovision software v4.8. For the evaluation of proliferation and survival the total number of BrdU+ cells in each hippocampal section (both hemispheres) was determined and a sum for each animal was calculated. Double-stainings in the survival paradigm were evaluated for the determination of cell differentiation based upon the number of cells co-expressing BrdU and either of the marker proteins (NeuN for mature neurons, GFAP for astrocytes). DCX stainings were used for the morphological characterization of newborn cells according to a previous study [31].

### Electrophysiological recordings

Long-term potentiation (LTP) and basal synaptic transmission (Input/Output (I/O) curve and paired-pulse inhibition (PPI)) were evaluated in acutely dissected hippocampal slices. The procedures for the hippocampal slice preparation and electrophysiological recordings followed previously published protocols with some minor modifications [32]. Briefly, mice were killed and hippocampi were dissected under the microscope. Hippocampi were cut into 400 μm thick slices using the McIlwain Tissue Chopper (Mickle Laboratory Engineering, Guildford, Surrey, UK) and kept in artificial-cerebrospinal fluid (aCSF, in mM: 125 NaCl, 2.5 KCl, 25 NaHCO3, 2 CaCl2, 1 MgCl2, 25 D-glucose, 1.25 NaH2PO4 with pH 7.4), that was continuously bubbled with a 95/5% mixture of O_2_/CO_2_ to maintain physiological conditions. Slices were recovered for at least 1 h at 32 °C before recordings. Same physiological conditions were provided in the recording chamber with exception that picrotoxin was added at concentration of 30 μM (Sigma-Aldrich, St. Louis, USA). Field excitatory postsynaptic potentials (fEPSPs) were recorded in the dentate gyrus (DG) with borosilicate glass pipette (2-5 MΩ), pre-filled with aCSF solution, after stimulation of the medial perforant pathway (MPP). The stimulating bipolar electrode was made of teflon-coated tungsten wire that had a 50 μm diameter and was located ~ 400 μm away from the recording electrode. The proper positioning of the electrodes was confirmed by applying a PPI protocol with 50 ms interstimulus interval at 30% of maximal voltage value that was determined by I/O curve. The PPI protocol consisted of a series of paired pulse stimulations with varying interstimulus intervals and used the ratio between second and first response as measurement. I/O curves were obtained by recording responses from repeated stimulation with pulses of voltage 1–9 V with interstimulus interval of 1 V. The decaying slopes of field potentials were normalized to the maximum value of the slope. LTP was induced by high frequency stimulation, consisting of 4 trains of 100 pulses at 100 Hz with 15 s inter-train intervals. Synaptic potentiation was determined by analyzing the changes in the decaying phase of the fEPSP slopes normalized to the baseline values. Data were recorded by an AxoClamp-2B amplifier (bridge mode) and a Digidata-1440 interface (Axon Instruments, Foster City, USA). pClamp-10 (Molecular Devices, Biberach, Germany) was used for offline analysis.

### RNA sequencing

After cervical dislocation, hippocampi were rapidly dissected on ice and total RNA was extracted using the miRNeasy mini kit (Qiagen, Hilden, Germany) according to the manufacturer’s instructions including the optional DNase digest step (Qiagen, Hilden, Germany). RNA-Seq libraries were prepared with the TruSeq Stranded mRNA LT sample preparation kit (Illumina, San Diego, CA, USA) using Sciclone and Zephyr liquid handling workstations (PerkinElmer, Waltham, MA, USA) for pre- and post-PCR steps, respectively. For sequencing, samples were diluted and pooled into NGS libraries in equimolar amounts. Expression profiling libraries were sequenced on a HiSeq 3000 instrument (Illumina, San Diego, CA, USA) in 50-base-pair, single-end mode. Transcriptome analyses were performed with the Tuxedo suite. For each sample, reads passing vendor quality filtering were aligned to the mm10 reference genome assembly provided by the UCSC Genome Browser based on Genome Reference Consortium GRCm38 with TopHat2 (v2.1.1, [33]). Cufflinks (v2.1.1, [34]) allowed for transcriptome assembly on the basis of the reference transcriptome and spliced read alignments, as well as raw transcript quantification. Cuffdiff was used for differential expression calling. Transcripts with a false discovery rate FDR ≤ 0.05 and a log_2_-fold change ≥ + 1.5 or ≤ − 1.5 for up- and downregulated genes, respectively, were considered as significantly differentially expressed transcripts (DETs).

Gene ontology enrichment analysis was performed for biological pathway, cellular compartment and molecular function sub-ontologies using the freely available software tools DAVID [35, 36] and Enrichr [37, 38].

### Neuropathohistology

Gross neuropathohistological assessment was carried out on coronal brain sections stained with hematoxylin/eosin (H&E) followed by immunohistochemical assays suitable for the examination of the integrity of neuronal cell bodies, dendrites and axons (anti-phosphorylated neurofilament-H, SMI31 and anti-non-phosphorylated neurofilament-H, SMI32). Briefly, after transcardial perfusion, frozen brains were cut on a brain slicer (Zivic Instruments, Pittsburgh, USA) into 2 mm sections and embedded into paraffin. The tissue blocks were cut into 4 μm slices and automated immunohistochemistry was performed on Autostainer 48 Link instruments (Dako, Glostrup, Denmark) using EnVision™ FLEX+ detection system (Dako, Glostrup, Denmark) according to the manufacturer’s recommendations. All primary antibodies were incubated for 30 min at room temperature using a dilution of 1:50000 for anti-SMI-31 (Biolegend, San Diego, USA) and 1:200 for anti-SMI-32 (Biolegend, San Diego, USA). All sections were counterstained with 3,3′-diaminobenzidine (DAB, Merck, Darmstadt, Germany) for specific labeling and Mayer’s hemalum solution (Merck, Darmstadt, Germany) for nuclear labeling. Images were scanned on a Pannoramic midi Scanner (3DHISTECH Ltd., Budapest, Hungary) and qualitatively analyzed using Pannoramic Viewer (3DHISTECH Ltd., Budapest, Hungary).

For the validation of myelin transcripts Mouse on Mouse Immunodection kit (MOM, Vector laboratories, Burlingame, USA) was used, employing the following primary antibodies: anti-MOG (myelin oligodendrocyte glycoprotein, Sigma-Aldrich, St. Louis, USA, 1:1000), anti-PLP1 (myelin proteolipid protein, BioRad, Hercules, USA, 1:500) and anti-CNP (2′,3′-Cyclic-nucleotide 3′-phosphodiesterase, Biolegend, San Diego, USA, 1:1000). The intensity of the DAB signal in MOG and PLP1 stainings was automatically determined in a selected region of interest within the hippocampus using Qupath [39]. For the number of oligodendrocytes, CNP+ cells were manually counted in a selected region of interest within the hippocampal dentate gyrus.

### Mitochondrial respiratory chain enzymatic activities

Enzymatic activities of mitochondrial respiratory complexes I – IV and citrate synthase (as reference enzyme) were tested using a standard published method [40]. Snap-frozen hippocampal tissue was homogenized with a glass-glass tissue grinder and centrifuged at 600G for 10 min at 4 °C to isolate mitochondria. Supernatant was aliquoted to avoid multiple freeze-thaw cycle. Total protein concentration was measured using Bradford assay (BioRad, Hercules, USA). Enzymatic activity measurements were conducted at a constant temperature of 37 °C using Shimadzu Spectrophotometry (UV-1800 Shimadzu, Kyoto, Japan).

### Mitochondrial membrane potential

Primary hippocampal neurons were prepared from postnatal day 1 WT and KI pups according to previously described protocol with some minor modifications [41]. Briefly, mouse hippocampi were isolated and cells were cultured at a density of 50,000 cells/cm^2^ on poly-D-lysine coated glass-bottom dishes (35 mm) using Neurobasal A medium supplemented with 2% B27, 0.5 mM glutamax and penicillin/streptomycin (10,000 U/ml). Cells were maintained for 2 weeks at 37 °C with 5% CO_2_.

The mitochondrial membrane potential (Ψ_m_) was measured using a fluorescence-based method as previously published with some adaptions in concentration and laser intensities [42]. Briefly, cells were equilibrated for 1 h at 37 °C in external buffer containing 1 nM TMRM to avoid fluorescence quenching. The same concentration of TMRM was present in all perfusing solutions. The external buffer contained: 140 NaCl, 3 KCl, 2 CaCl2, 2 MgCl2, 10 HEPES, 20 glucose (in mM, pH 7.4). All chemicals were purchased from Thermofisher Scientific, Waltham, USA. Pharmacological intervention with ATP synthase inhibitor (Oligomycin), complex III inhibitor (Antimycin A) and protonophore (FCCP) were made (all substances ordered from Sigma-Aldrich, St. Louis, USA).

After loading, cells were imaged using a confocal microscope (Nikon A1r, Minato, Japan) by excitation/emission wavelengths of 562 nm/595 nm. Experiments were performed at room temperature and cells were continuously locally superfused using an 8-reservoir drug application system (Octaflow II). Image analysis was conducted by specifying mitochondria located in neuronal bodies as region of interest and the mean fluorescence intensity profile over time was calculated. All images were quantified using open access ImageJ (Fiji) [43].

### Statistical analyses

Sample sizes were determined according to previous studies [24, 44, 45]. All data were tested for normality using the Kolmogorov-Smirnov test prior to further statistical evaluation. Cosinor analysis was performed to determine circadian oscillations. Data were analyzed using CircWave statistic software (V1.4) according to the eq. Y = c + aSIN(i2π *t*/24) + bCOS(i2π *t*/24), where Y is delta cycle threshold, *t* is circadian time, and a, b, and c were predicted. Further nonlinear regression analysis was used to determine peak time, trough and amplitude of the curve, using the center of gravity parameter. Behavioral experiments were analyzed using 2-way ANOVA, repeated measure ANOVA and 3-way ANOVA. Neurogenesis and mitochondrial results were evaluated by Student’s t-test or corrected with Welch’s t-test, where appropriate. Electrophysiological data were analyzed using repeated measure ANOVA. Statistical outliers (values outside of the interval: mean ± 2 standard deviations) were excluded from further analyses. The threshold for significance was set at *p < 0.05 in all instances. Statistical analyses were performed using SPSS software for Windows, Version 24 (IBM Corporation, Chicago, USA) and Graphpad Prism, Version 7 (San Diego, USA).

## Results

### Characterization of emotional behavior in KI mice

**Fig. 1:**
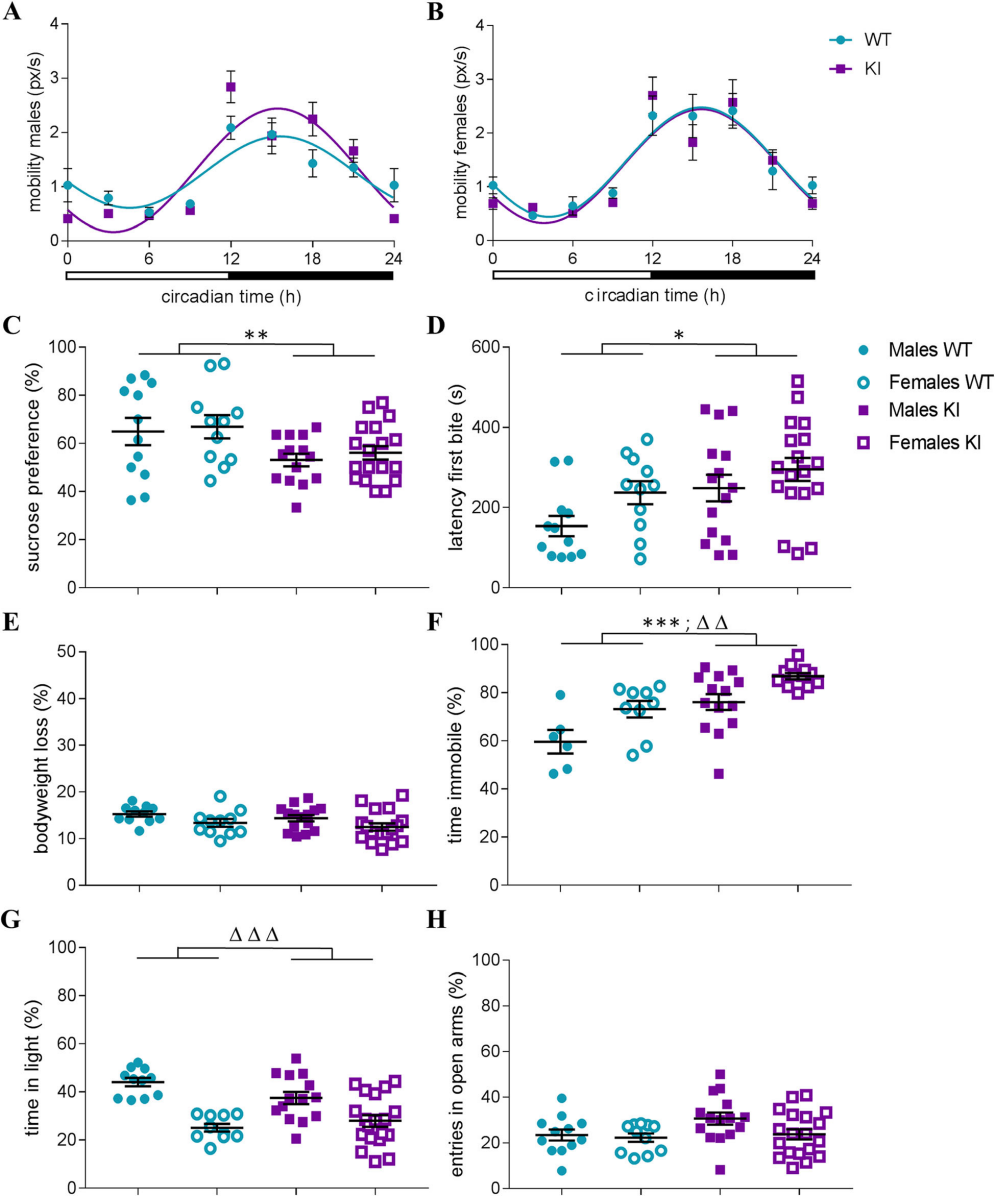
Behavioral characterization of HMBS-deficient mice. 24 h mobility patterns in **a** male and **b** female HMBS-deficient (KI) and wildtype (WT) control mice. Assessment of depression-like behavior in **c** SPT (% sucrose preference), **d** NSF (latency until first bite) without alterations in **e** body weight loss during food deprivation. **f** Behavioral despair in the TST (% of time spent immobile). Examination of anxiety-like behavior in **g** LD (% of time in light compartment) and **h** EPM (% of entries into open arms). All values are presented as mean ± SEM; *n* = 6–19 per group. Statistical significances displayed refer to genotype effects (*) and sex effects (∆) resulting from repeated measure and 2-way ANOVA. *p < 0.05, **p < 0.01, ***p < 0.001; HMBS, hydroxymethylbilane synthase; SPT, sucrose preference test; NSF, novelty suppressed feeding; TST, tail suspension test; LD, light/dark box; EPM, elevated plus maze

We initially analyzed the behavioral phenotype of HD-AIP mice comparing adult male and female KI animals to wildtype littermate controls, focusing on depression- and anxiety-like displays, in light of the symptoms reported in AIP patients. In consideration of the previously described motoric deficiencies in the KI mice, particularly during the neonatal and early postnatal period [13], we first examined overall locomotor activity in adult animals to exclude a possible bias in the subsequent behavioral paradigms. Long-term (24 h) assessment of home cage activity revealed no genotype effect on total mobility in male and female KI mice (Fig. 1a-b). Subsequent cosinor analysis documented statistically significant 24 h rhythmic patterns of mobility for all groups (*p* < 0.0001). These data excluded locomotor impairment as a liability for the behavioral assessment of adult KI mice and allowed us to proceed to mood-specific tests.

Male and female KI mice showed reduced sucrose preference compared to their respective controls in the SPT, indicating augmented depression-related anhedonic behavior (Fig. 1c; significant main effect of genotype (F(1,51) = 8.37, *p* < 0.01). In line with this observation, enhanced depression-related anxiety in the KI mice was revealed in the NSF test as evidenced by a longer latency to the first bite (Fig. 1d; significant main effect of genotype (F(1,52) = 6.24, *p* < 0.05). Importantly, no significant differences between genotypes were observed in the amount of bodyweight loss after a 24 h food restriction (Fig. 1e). Furthermore, there was no correlation between home cage food consumption (5 min after the NSF) and the latency to the first bite in the NSF (*R*^*2*^ = 0.048 for males, *R*^*2*^ = 0.076 for females), collectively excluding an unspecific bias in the NSF due to metabolic alterations and/or motivation to eat. In agreement with the previous results, immobility in the TST, commonly used as an indicator for behavioral despair in the context of depression-like behavior, was elevated in male and female KI mice (Fig. 1f; significant main effect of genotype (F(1,37) = 20.62, *p* < 0.0001); significant main effect of sex (F(1,37) = 13.31, *p* < 0.001)). No alterations in anxiety-like behavior were detected in the KI mice in comparison to WT controls in the LD (time in the light zone) or the EPM (open arm entries). A significant main effect of sex (F(1,50) = 35.05, *p* < 0.0001) for the time spent in light compartment was observed in the LD (Fig. 1g-h).

In the TST, depression-like behavior was reduced by acute treatment with the selective serotonin reuptake inhibitor (SSRI) Escitalopram (10 mg/mg i.p.) in mice of both genotypes (reduction in % immobility (mean ± SEM: KI males 78.44 ± 2.56% to 43.60 ± 6.19%; KI females 86.84 ± 1.14%; WT males to 53.00 ± 8.61%; WT males 59.63 ± 4.00% to 23.13 ± 3.72%; WT females: 73.12 ± 4.66% to 44.13 ± 7.38%).

Since no sex × genotype interactions were observed for any of the behavioral tests, samples were collapsed across sexes for all following experiments. The number of animals of each sex used for the individual analyses as well as total sample sizes are summarized in Additional file 1.

### Myelination related transcripts are differentially expressed in the hippocampus of KI mice

Based on the depression-like behavior detected in the KI mice, our subsequent analyses focused on the hippocampus, a limbic brain structure central to the pathophysiology of depression [46–48].

We performed high-throughput RNA sequencing (RNA-Seq) in order to explore the molecular signature accompanying the behavioral deficits in the KI mice in an unbiased manner. Three hundred twenty transcripts were found to be significantly and more than 1.5-fold differentially expressed between genotypes. A list of all differentially expressed genes (DEGs) is provided in Additional file 2. Raw data are available at www.ncbi.nlm.nih.gov/geo/query/acc.cgi?acc=GSE146212.

**Fig. 2:**
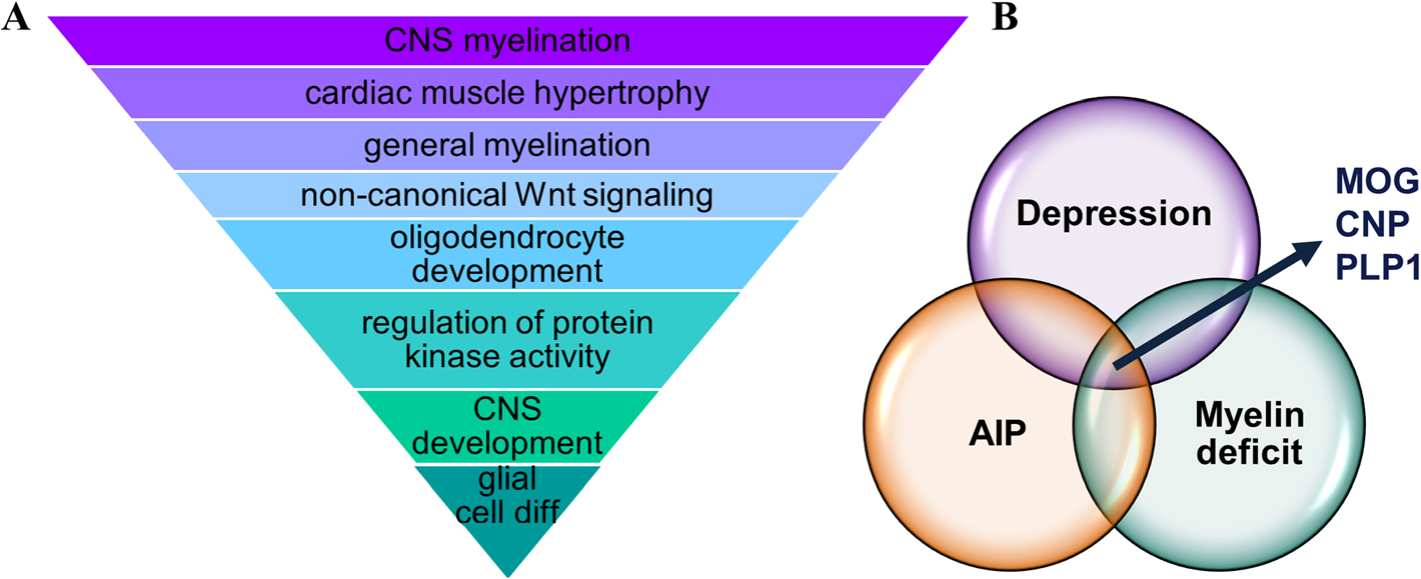
High-throughput RNA sequencing of hippocampal tissue of HMBS-deficient mice. **a** Results of enrichment analysis revealing a gene expression signature with relevance for myelination in KI mice. **b** Venn-diagram highlighting 3 genes found to be differentially expressed in KI as compared to WT mice which are also involved in depression and myelin deficits (*n* = 6 per genotype). AIP, acute intermittent porphyria; MOG, myelin oligodendrocyte glycoprotein; CNP, 2′,3′-Cyclic-nucleotide 3′-phosphodiesterase; PLP1, myelin proteolipid protein 1 (CNS)

Bioinformatic analyses focusing on biological pathways, cellular compartments and molecular function sub-ontologies revealed a notable enrichment of DEGs for CNS myelination and oligodendrocyte development (Fig. 2a). To gain further insight into the possible pathophysiological implications of the molecular alterations in the KI hippocampus, differentially expressed genes in KI mice were contrasted against available gene sets from DisGeNet [49] with relevance for depression and myelin deficits. Three genes were detected in the three-way comparison: Myelin Oligodendrocytes Glycoprotein (MOG), 2′,3′-Cyclic-nucleotide 3′-phosphodiesterase (CNP) and Proteolipid Protein 1 (PLP1) (Fig. 2b).

### KI mice present with defective myelination and a reduction in oligodendrocytes number

**Fig. 3:**
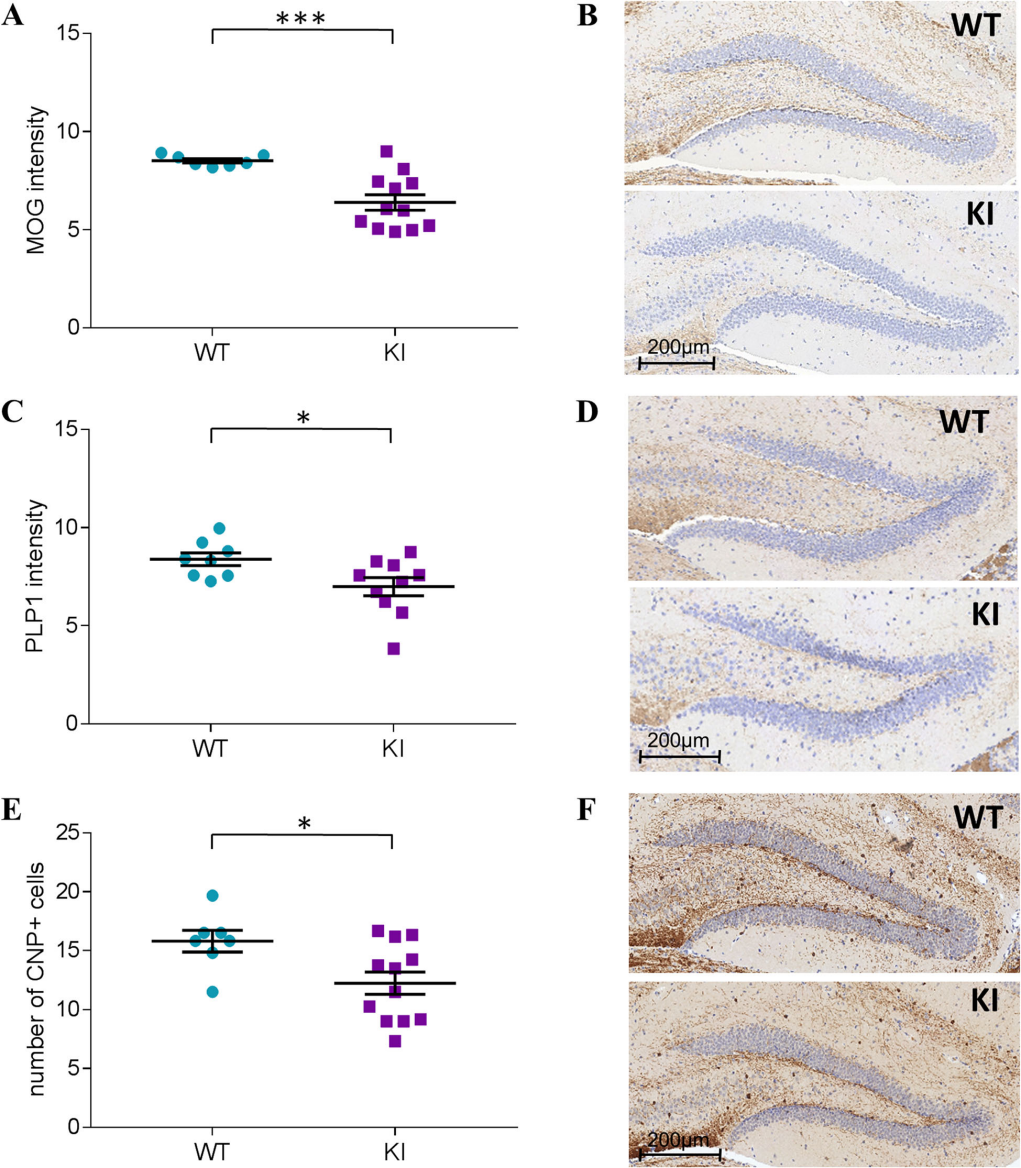
Immunohistochemical evaluation of myelin proteins in the hippocampus of HMBS-deficient mice. **a** Evaluation of MOG intensity and **b** microscopal image of MOG staining in KI and WT mice, **c** PLP1 intensity and **d** microscopal image of PLP1 staining in KI and WT mice. **e** Number of CNP+ cells and **f** microscopal image of CNP staining in KI and WT mice. All values are presented as mean ± SEM; *n* = 7–12 per genotype/3 sections per mouse. Statistical significances displayed are results of Student’s t-test. *p < 0.05, ***p < 0.001; HMBS, hydroxymethylbilane synthase; MOG, myelin oligodendrocyte glycoprotein; PLP1, myelin proteolipid protein 1; CNP, 2′,3′-Cyclic-nucleotide 3′-phosphodiesterase

In order to validate and extend the RNA-Seq results, hippocampal protein levels of MOG, PLP-1 and CNP were examined immunohistochemically. In accordance with the results at the mRNA level, the intensity of the 3,3′-Diaminobenzidine (DAB) staining revealed a significant decrease in MOG (Fig. 3a-b; t(12.52) = 5.18, *p* < 0.001) and PLP1 (t(16) = 2.35, *p* < 0.05) protein expression in KI mice (Fig. 3c-d). A significant difference between genotypes was also observed for the number of CNP+ cells (Fig. 3e-f; t(17) = 2.49, *p* < 0.05), indicating fewer oligodendrocytes in KI than in control littermates.

### Altered mitochondrial activity in KI mice

Subsequent efforts explored mitochondrial dysfunction as a possible pathophysiologic link between HMBS-deficiency and its impact on affective behavior and myelination. ALA has been previously shown to detrimentally impact myelination in the peripheral nervous system, and this effect was proposed to result from mitochondrial respiratory chain defects and ensuing oxidative damage [50, 51].

**Fig. 4:**
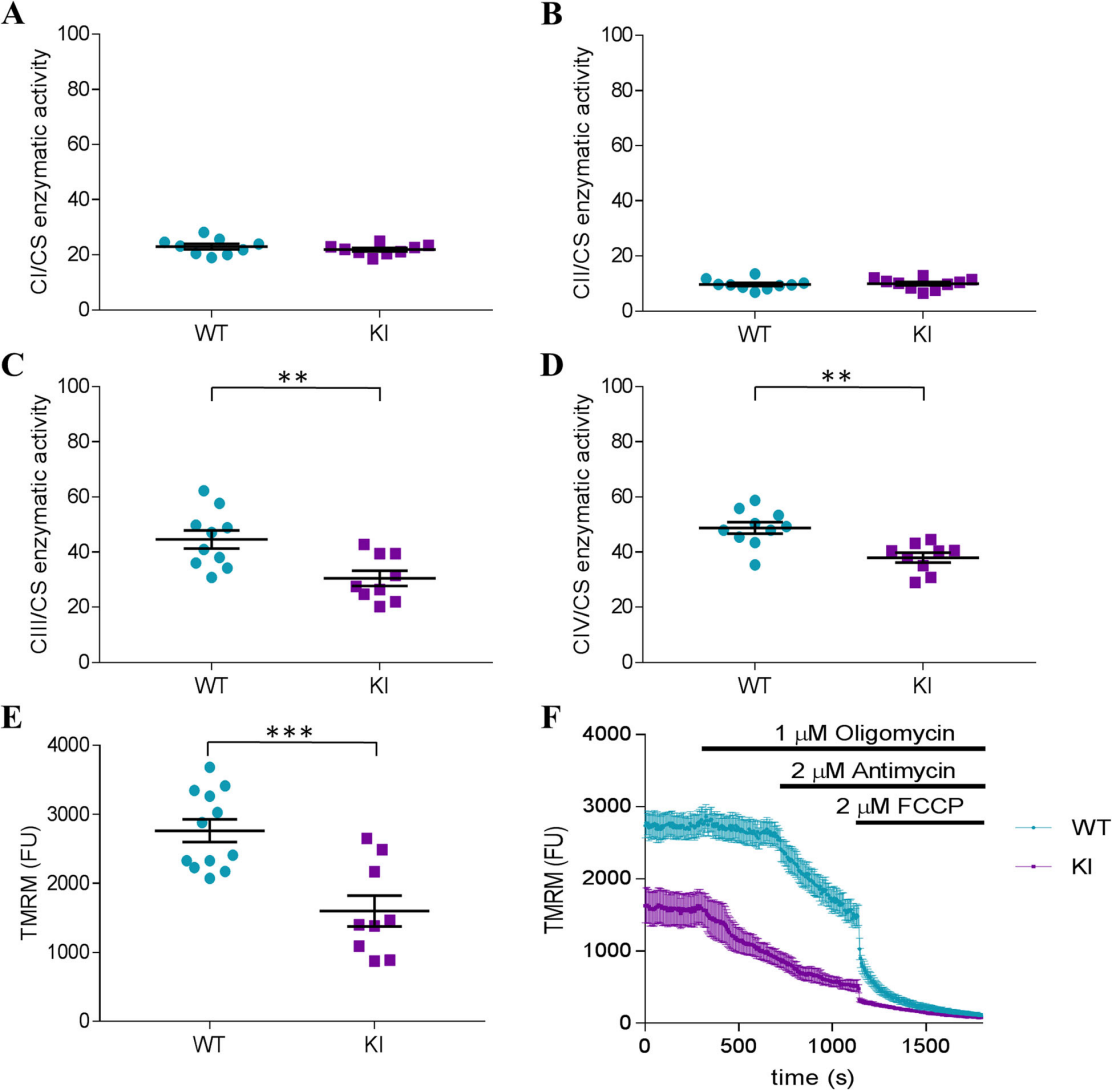
Mitochondrial activity and membrane potential in HMBS-deficient mice. Measurement of the relative enzymatic activity (ratio to citrate synthase as housekeeping enzyme) of the four respiratory chain complexes in the hippocampus in **a** CI, **b** CII, **c** CIII and **d** CIV; **a-d***n* = 9–10 per genotype. TMRM assay of **e** the basal TMRM fluorescence in cultured hippocampal neurons obtained from WT mice and KI mice. **f** Response of TMRM fluorescence to ATP synthase inhibitor Oligomycin, complex III inhibitor Antimycin A and protonophore FCCP in primary hippocampal neurons of KI and WT mice; **e-f***n* = 9–12 per genotype. All values are presented as mean ± SEM. Statistical significances displayed are results of Student’s t-test. **p < 0.01, ***p < 0.001; HMBS, hydroxymethylbilane synthase; CI-IV, mitochondrial complex I-IV, TMRM, tetramethyl rhodamine ethyl ester, FCCP, carbonyl cyanide-4-(trifluoromethoxy)phenylhydrazone

We therefore determined the activity of the four mitochondrial respiratory chain complexes (CI-CIV) individually in mitochondria isolated from KI and WT hippocampal tissue using citrate synthase as a quantitative marker enzyme. No significant difference between genotypes for CI (NADH:ubiquinone oxidoreductase) and CII (succinate dehydrogenase) activities were found (Fig. 4a and b). However, for the heme containing complexes CIII (cytochrome c reductase) and CIV (cytochrome c oxidase) (Fig. 4c and d), a significant decrease of enzymatic activity (t(17) = 3.257, *p* < 0.01 and t(17) = 3.867, *p* < 0.01) in KI mice compared to WT littermates was revealed.

Reduced activity of these respiratory complexes may result in lower efficiency of mitochondrial respiration. Therefore, we measured mitochondrial membrane potential (Ψ_m_), which is directly controlled by the activity of respiratory chain complexes and the activity of mitochondrial ATP synthase, in-vitro using primary hippocampal neurons from KI and WT mice. Mitochondrial function is coupled to the state of polarization [52] and indeed our data demonstrate that mitochondria of KI neurons were less polarized (lower TMRM intensity) compared to those of WT neurons (Fig. 4e; t(19) = 4.29, *p* < 0.001). In the next step we examined ψm maintenance. Mitochondria with normal respiratory activity maintain the ψm by the proton pumping activity of the respiratory chain while ATP synthase couples a depolarizing proton influx to the ATP synthesis. However, in the case of reduced respiratory function, ATP synthase may reverse its mode of operation and pump protons out of the mitochondrial matrix, thus maintaining ψm at the expense of ATP. Incubation with the ATP-synthase inhibitor Oligomycin (1 μM) did not change the TMRM fluorescence in WT mitochondria, while the subsequent application of the complex III inhibitor Antimycin A (2 μM), led to the expected gradual decrease of TMRM fluorescence, indicating that in the mitochondria of WT neurons, Ψ_m_ is maintained by the activity of respiratory chain (Fig. 4f). In contrast, addition of Oligomycin resulted in a decrease of TMRM fluorescence in the mitochondria of KI neurons, while the subsequent addition of Antimycin A exerted only a modulatory effect on the ongoing depolarization. Protonophore FCCP (2 μM) was added to achieve a maximal depolarization at the end of the experiments in order to demonstrate that the measured fluorescence originated from mitochondria. See Additional file 3 for the confirmation of the localization of TMRM in mitochondria in original micrographs.

### Adult hippocampal cell proliferation and differentiation is impaired in KI mice

To further explore the consequences of disrupted mitochondrial energy metabolism on hippocampal function, we focused on its neurogenic and neuroplastic potentials, both of which have been also repeatedly linked to depression-like behavior.

**Fig. 5:**
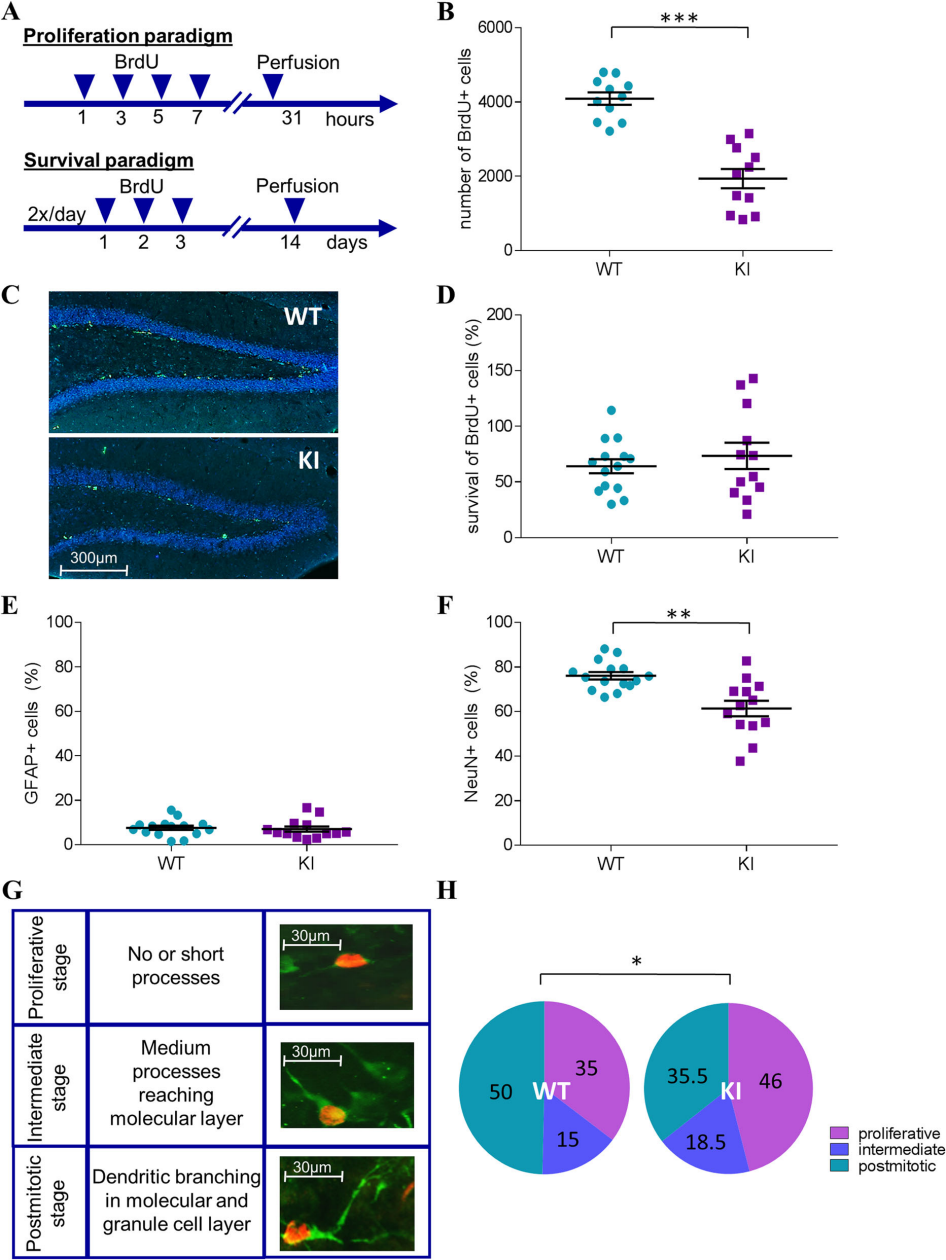
Analysis of adult hippocampal neurogenesis in HMBS-deficient mice. **a** BrdU injection schedules for the proliferation and survival paradigms. **b** Quantification of hippocampal progenitor cell proliferation (BrdU+ cell count) and **c** respective fluorescence microscope images in HMBS-deficient (KI) and wildtype (WT) mice (BrdU+ cells in green). **d** Survival of newborn (BrdU+) cells over a two-week period. **e** Relative differentiation rates of newborn (BrdU+ cells) cells into astrocytes (GFAP+ cells) and **f** mature neurons (NeuN+ cells). **g** Developmental stages of doublecortin stained DCX+ (green) BrdU+ (red) labeled cells according to defined morphological criteria (proliferative, intermediate and postmitotic). **h** Quantification (%) of BrdU+ cells in each developmental stage in KI and WT mice. All values are presented as mean ± SEM; *n* = 11–13 per genotype/3 sections per mouse. Statistical significances displayed are results of Student’s t-tests. ***p < 0.001, **p < 0.01, *p < 0.05; HMBS, hydroxymethylbilane synthase; BrdU, 5-bromo-2′-deoxyuridine; GFAP, glial fibrillary acidic protein; NeuN, neuronal nuclei; DCX, doublecortin

BrdU administration in the proliferation and survival paradigms was used to investigate dentate gyrus neurogenesis (Fig. 5a). Quantification of BrdU+ cells, revealed a highly significant reduction in hippocampal progenitor cell proliferation in the KI mice compared to WT littermates (Fig. 5b-c; t(20) = 6.98, *p* < 0.001). However, the rate of survival of newborn cells was comparable among genotypes (Fig. 5d). Subsequent co-staining with astrocytic (GFAP) and neuronal (NeuN) markers for the evaluation of cell fate demonstrated no difference in the relative amount of cells differentiated into astrocytes (Fig. 5e), but a significant decrease (t(17.25) = 3.80, *p* < 0.01) in mature neurons among all BrdU+ cells in the KI mice was observed (Fig. 5f; NeuN+ cells co-labeled with BrdU).

To gain deeper insight into the process of neuronal differentiation in adult-born cells, morphologically distinct stages of neural maturation [31] were examined using doublecortin (DCX) stainings in BrdU+ cells (Fig. 5g). Here, a selective deficit in the differentiation into fully mature, postmitotic neurons was detected in the KI mice, as indicated by a higher percentage of BrdU+ cells in the early proliferative (t(14.23) = 2.64, *p* < 0.05) and intermediate phases (t(25) = 2.25, *p* < 0.05) and a lesser percentage in the postmitotic stage (t(12.42) = 2.70, *p* < 0.05) than in WT littermates (Fig. 5h).

To further explore the consequences of disrupted mitochondrial energy metabolism on hippocampal function, we focused on its neurogenic and neuroplastic potentials, both of which have been also repeatedly linked to depression-like behavior.

### Aberrant synaptic plasticity parallels behavioral and neurogenic deficits in KI mice

**Fig. 6:**
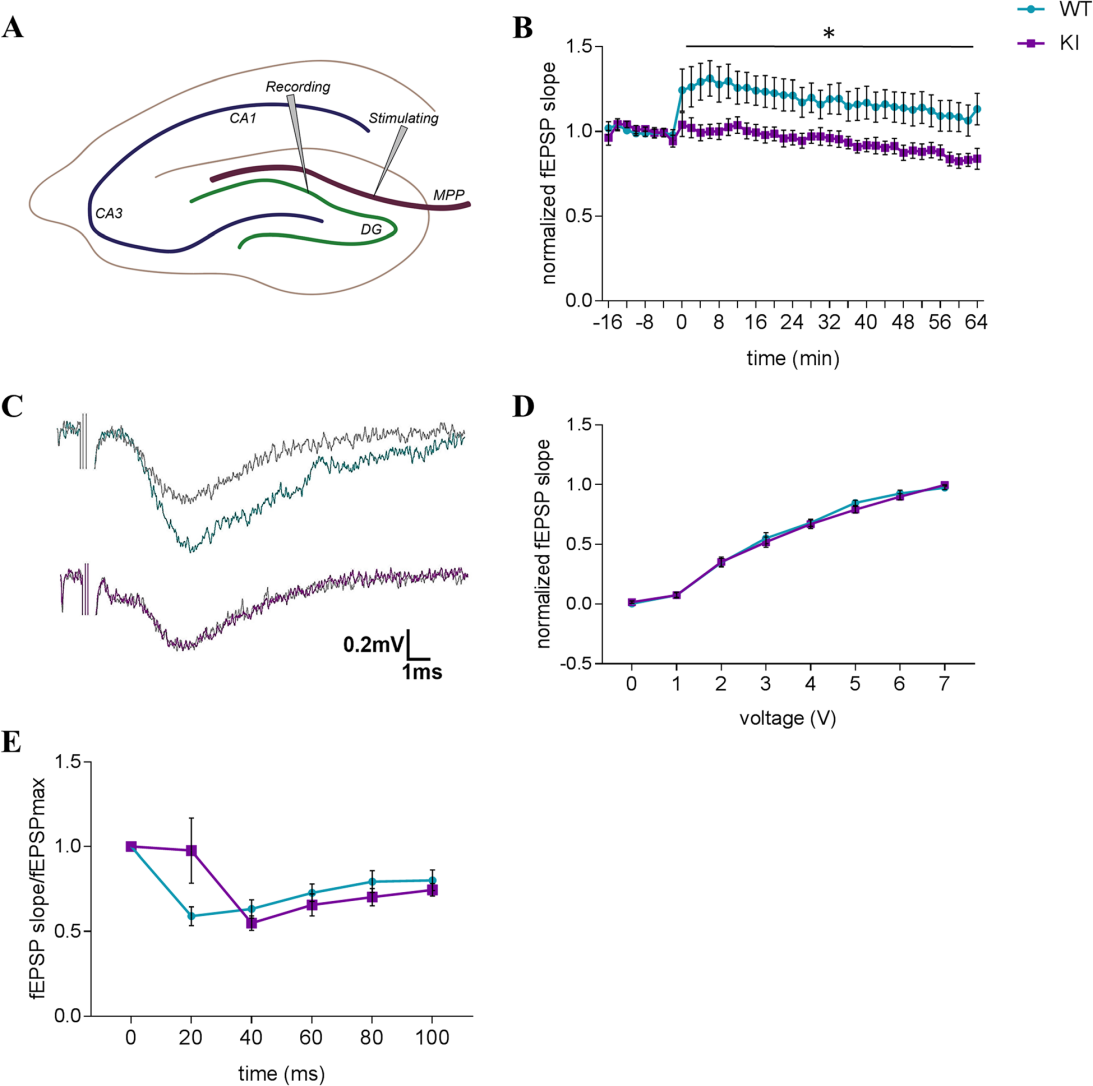
Electrophysiological evaluation of hippocampal dentate gyrus synaptic plasticity in HMBS-deficient mice. **a** Schematic depiction of the placement of the electrodes for LTP recordings. **b** LTP of fEPSP after high frequency stimulation and **c** representative traces in HMBS-deficient (KI) and WT mice. **d** Input-output recordings and **e** paired-pulse inhibition of fEPSP to maximum fEPSP. All values are presented as mean ± SEM; *n* = 8–12 per genotype. Statistical significances displayed reflect results of repeated measure mixed model ANOVA. *p < 0.05; HMBS, hydroxymethylbilane synthase; LTP, long-term potentiation; fEPSP, field excitatory postsynaptic potential, MPP, medial perforant path; DG, dentate gyrus

We next interrogated hippocampal synaptic plasticity by assessing long-term potentiation as a form of activity-dependent enhancement of synaptic strength. We found that stimulation of the medial perforant path (Fig. 6a) resulted in a significantly blunted response (i.e., potentiation) in dentate gyrus to high frequency stimulation (HFS) in the KI mice compared to WT littermate controls (Fig. 6b and c; F(1,40) = 4.46, *p* < 0.05).

Basal synaptic function was intact in the KI hippocampus, as reflected in comparable slopes of input-output recordings between genotypes (Fig. 6d). We further found the alterations in synaptic plasticity in the KI mice to be primarily relevant for long-term synaptic plasticity, as no genotype effect was notable in paired-pulse inhibition recordings, representative of short-term plasticity events (Fig. 6e).

Using immunohistochemical stainings for standard markers for neuronal integrity (H&E, SMI-31 and SMI-32), gross neuropathohistological defects were excluded as non-specific biases of the electrophysiological and behavioral results (Additional file 4).

## Discussion

In a comprehensive characterization of the HD-AIP mouse model we revealed, for the first time, augmented depression-like behavior as a consequence of severe, constitutive HMBS deficiency. This phenotype was confirmed using three independent paradigms routinely employed for the evaluation of depression-like behavior in mice, excluding task-specific performance as artifacts and highlighting the validity of the findings. Importantly, augmented immobility in the TST was reduced by acute administration of Escitalopram, suggesting SSRI treatment as possible therapeutic strategy for AIP patients suffering from affective symptomatology. Along these lines it will be interesting to explore in future studies whether and how the behavioral consequences of severe HMBS deficiency interact with the effects of acute or chronic stress exposure and the related sensitivity to pharmacotherapy.

As previous study revealed motor deficits of the KI mice [13], a possible bias as a result of hypolocomotion was ruled out by 24 h continuous activity monitoring. Considering that psychiatric manifestations are often associated with dysregulation of circadian rhythms [53] it is noteworthy that the circadian rhythmicity of daily activity was preserved in KI mice.

Interestingly, despite the robust augmentation of depression-like behavior in the KI mice, no effect on anxiety-like behavior was observed. One can speculate that the brain regions more relevant to the neural circuitry of depression than of anxiety are specifically vulnerable to the pathophysiological sequelae of accumulation of ALA and PBG. This consideration led us to focus our subsequent interrogation on the hippocampus, due to its relevance for the pathophysiology of depression [46–48]. However, we cannot exclude that other brain regions may also be affected/ involved. We employed RNA-Seq analysis as hypothesis-free approach to explore the molecular consequences of severe HMBS-deficiency in the KI hippocampus. Bioinformatic workup of differentially expressed transcripts revealed impaired myelination and oligodendrocyte development emerged as relevant biological theme. When we specifically analyzed differential gene expression in KI mice, genes that have been previously linked to myelination deficits and depression-like states, MOG, PLP1 (myelin markers) and CNP (oligodendrocytic marker) were implicated as molecular players. Subsequent immunohistochemical analyses corroborated these findings at the protein level, further supporting the role of defective myelination as a potential pathogenic mechanism in the behavioral, neurogenic and plasticity defects in KI mice. Indeed, evidence from previous animal studies, post-mortem human studies, as well as genetic and neuroimaging experiments indicate an association between major depressive disorder (MDD) and changes in brain myelin content [54]. Interestingly, patients with neurological disorders of the white matter, like multiple sclerosis (MS) often show psychiatric symptoms, including depression, and display comparable dysregulation in gene expression [55, 56]. In line with our observations, myelin deficits have been revealed in the seminal characterization of the (HD-AIP) KI mice [13] and have also been reported in AIP patients [57]. However, the mechanism by which HMBS deficiency may alter CNS myelination has remained elusive to date.

Disturbed mitochondrial energy production is frequently observed in congenital diseases and often related to neurological and psychiatric symptoms [58, 59]. Taking into consideration that half of the heme biosynthetic pathway is processed in the mitochondria and that disturbed heme metabolism causes mitochondrial defects [50, 51], a possible direct link between AIP and mitochondrial dysfunction is plausible. Indeed, impairments of mitochondrial energy metabolism in the liver, muscle and brain have also been demonstrated in the T1/T2 mouse model following phenobarbital administration [50, 51]. We tested the impact of HMBS deficiency on mitochondrial function in the KI mouse model, selectively focusing on the hippocampus. While enzymes of the mitochondrial complexes CI and CII had comparable activity among genotypes, there was a significant decrease of enzymatic activity in complexes CIII and CIV in the KI mice compared to controls. Involvement of heme in the function of CII has been proposed, however its role is unclear to date. For CIII and CIV, the relevance of heme for electron transport and reduction of molecular oxygen, respectively, have been delineated in detail (for review see [60]). Hence, reduced heme availability in HMBS-deficient mice is likely to cause a selective impact on CIII and CIV activity.

The reduction in the enzymatic activity of the respiratory chain in hippocampal tissue was strengthened by the result of decreased mitochondrial membrane potential in primary hippocampal KI neurons. These results further indicate that the KI mitochondria were characterized by insufficient respiratory activity and maintained their membrane potential by reverse ATP synthase, resulting in ATP consumption rather than production. The reversal potential of ATP synthase activity is typically only a few mV below the resting mitochondrial membrane potential. Thus, even a mild decrease of mitochondrial membrane potential may be sufficient to reverse the action of ATP synthase [61].

To determine the consequences of disrupted mitochondrial energy metabolism on hippocampal function, we analyzed dentate gyrus neurogenesis and synaptic plasticity. Neurogenic aberrations have been reported in a variety of different animal models of depression [46–48, 62]. Proliferating and newly born cells are more susceptible to the lack of energy supply resulting from impaired mitochondrial function (for review see [63]). Along these lines we here found an impact of HMBS-deficiency specifically on dividing progenitor cells but not on postmitotic neurons or other cell types, as no gross histopathological alterations were observed in the KI hippocampus. Mechanisms of synaptic plasticity, including LTP, are also critically dependent on mitochondrial function (for review see [64]) and are deranged in several animal models of depression [65, 66]. Indeed, we here observed impaired activity-dependent synaptic strengthening in the KI dentate gyrus, which may be caused by mitochondrial dysfunction.

**Fig. 7:**
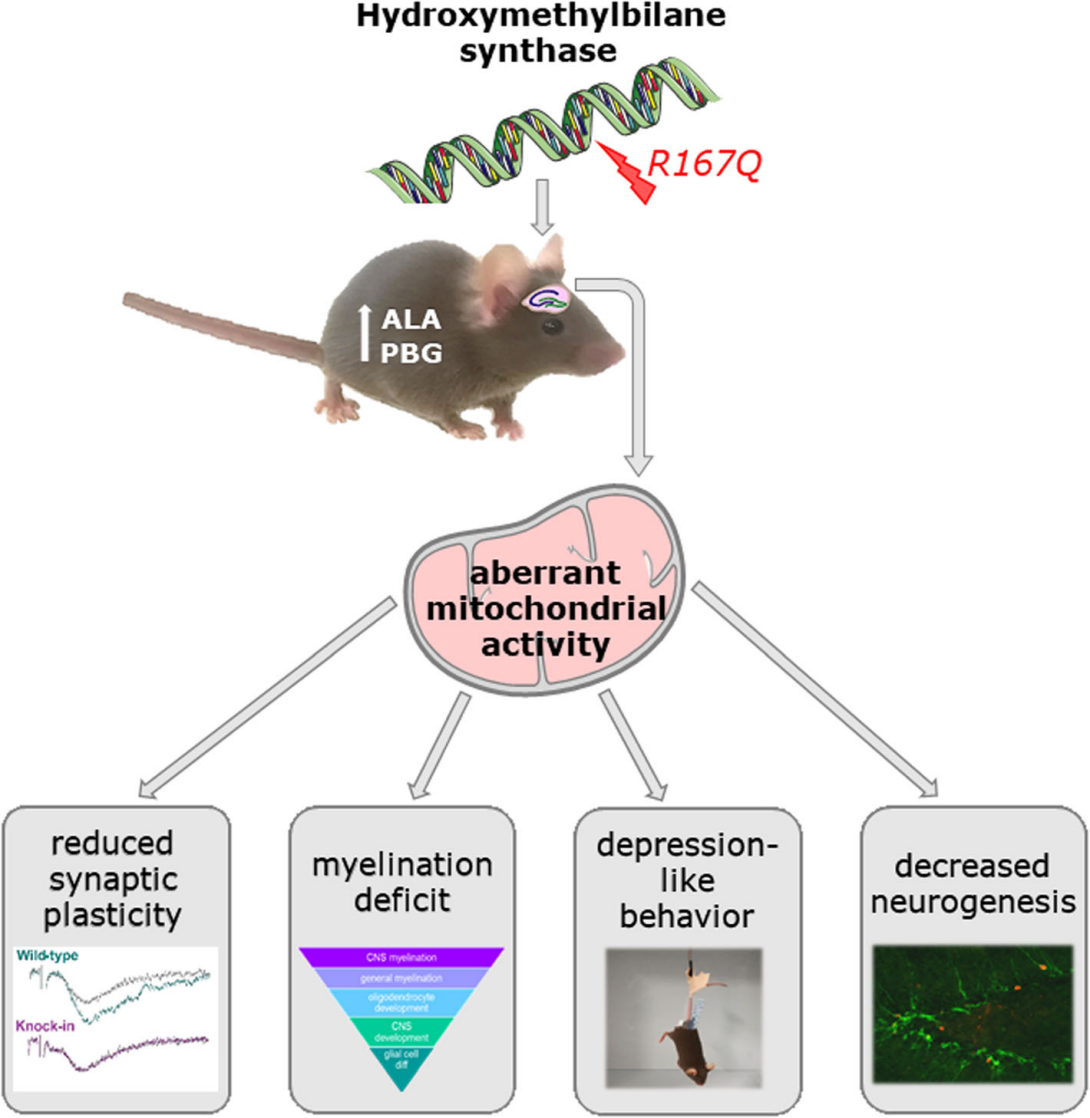
Model for the pathomechanisms involved in augmented depression-like behavior in HMBS-deficient mice. Aberrant mitochondrial activity is proposed as central pathological feature impacting on neurogenic and neuroplastic potential, as well as on myelination in the KI hippocampus, all of which may collectively contributed to enhanced depression-like behavior in HMBS-deficient mice

Aberrant mitochondrial function is increasingly recognized as an important element in the pathophysiological mechanisms of depression (for review see [59]) and physiological mitochondrial activity is pivotal for oligodendrocyte function and myelin production [67, 68]. These considerations close the loop to the affective and myelination deficits in KI mice and collectively suggest compromised mitochondrial function as pathogenetic mechanism contributing to the behavioral and neuropathological manifestations in the HMBS-deficient mouse model of HD-AIP (Fig. 7).

Thus, while HD-AIP and AIP are distinct metabolic disorders, our KI mice and phenobarbital-induced T1/T2 mice both display significant mitochondrial energetic defects in their CNS tissues. By extension, we propose that defective mitochondrial function may also contribute to the neuropsychiatric abnormalities that heterozygous AIP patients experience during acute attacks and should be considered for future development of preventive and therapeutic strategies for the clinical management of the disease.

## Conclusion

This study for the first time reveals the behavioral consequences of severe HMBS deficiency in a mouse model of homozygous-dominant AIP and proposes mitochondrial dysfunction as relevant pathophysiological mechanism.

## Supplementary information

**Additional file 1: Table S1.** Description of samples. Samples sizes (with sex and genotype) are reported according to experimental procedure.

**Additional file 2: Table S2.** 228 differentially expressed transcripts were downregulated in HMBS-deficient mice. Columns are as follows: Gene short-name, locus, sample 1+2, value 1+2 (mean reads RPKM), fold change, *p*-value, *q*-value. **Table S3.** 92 differentially expressed transcripts were upregulated in HMBS-deficient mice. Columns are as follows: Gene short-name, locus, sample 1+2, value 1+2 (mean reads RPKM), fold change, *p*-value, *q*-value.

**Additional file 3: Figure S1.** Basal TMRM fluorescence in cultured hippocampal neurons and pharmacological intervention with ATP synthase inhibitor (Oligomycin), complex III inhibitor (Antimycin A) and protonophore FCCP in KI and WT mice; *n* = 5–9 cells per genotype. TMRM, tetramethyl rhodamine ethyl ester, FCCP, carbonyl cyanide-4-(trifluoromethoxy)phenylhydrazone.

**Additional file 4: Figure S2.** Neuropathohistological evaluation of hippocampal coronal sections stained with hematoxylin-eosin (H&E), SMI-31 (phosphorylated epitope in neurofilament) and SMI-32 (non-phosphorylated epitope in neurofilament) of HMBS-deficient (KI) and wildtype (WT) mice; *n* = 8–12 per genotype.

## Data Availability

The datasets used and analyzed during the current study are available from the corresponding author on reasonable request and raw data of differentially expressed genes are available at www.ncbi.nlm.nih.gov/geo/query/acc.cgi?acc=GSE146212.

## Abbreviations

AIP: Acute Intermittent Porphyria
ALA: Aminolevulinc acid
ATP: Adenosinetriphosphat
CI-V: Complex I-V
CNP: 2′,3′-Cyclic-nucleotide 3′-phosphodiesterase
DAB: 3,3′-diaminobenzidine
DCX: Doublecortin
DG: Dentate gyrus
EPM: Elevated plus maze
FCCP: Carbonyl cyanide-p-trifluoromethoxyphenylhydrazone
FDR: False discovery rate
GFAP: Glial fibrillary acidic protein
HD: Homozygous dominant
HE: Hematoxylin-eosin
HMBS: Hydroxymethylbilane synthase
KI: Knock in
LD: Light dark
LTP: Long-term potentiation
MDD: Major depressive disorder
MOG: Myelin oligodendrocytic protein
MOM: Mouse on mouse
MPP: Medial perforant path
MS: Multiple sclerosis
NADH: Nicotinamide adenine dinucleotide hydrogen
NSF: Novelty suppressed feeding
PBG: Phorphobilinogen
PCR: Polymerase chain reaction
PFA: Paraformaldehyd
PLP: Proteolipid protein
PPI: Paired pulse inhibition
RL: Radioligand
RNA: Ribonucleic acid
SEM: Standard error of mean
SPT: Sucrose preference test
TMRM: Tetramethyl-rhodamine-methyl-ester
TST: Tail suspension test
WT: Wildtype
