# Supplementary material for: Severe hydroxymethylbilane synthase deficiency causes depression-like behavior and mitochondrial dysfunction in a mouse model of homozygous dominant acute intermittent porphyria
Source: Acta Neuropathol Commun. 2020 Mar 20;8:38. doi: 10.1186/s40478-020-00910-z (PMC7082933; doi:10.1186/s40478-020-00910-z)
Supplement: Supplementary file 1 — Additional file 1: Table S1. Description of samples. Samples sizes (with sex and genotype) are reported according to experimental procedure. [file 40478_2020_910_MOESM1_ESM.pdf]

Supplementary Table-1.

Experiment	Figure	Sample size					
		WT (control)			KI (HMBS-deficient)		
		sex	genotype	condition	sex	genotype	condition
**Phenotyper**	1A-B	6	12	in vivo	6	12	in vivo
**SPT**	1C	11-12	23	in vivo	15-19	34	in vivo
**NSF**	1D	11-12	23	in vivo	15-19	34	in vivo
**TST**	1F	6-9	15	in vivo	13-14	27	in vivo
**LD**	1G	11-12	23	in vivo	15-19	34	in vivo
**EPM**	1H	11-12	23	in vivo	15-19	34	in vivo
**RNAseq**	2A-B	6 males	6	hippocampus	6 males	6	hippocampus
**Myelin IHC**	3A-F	4-6	10	sections	6	12	sections
**Mitochondria enzyme activity**	4A-D	4-6	10	hippocampus	4-6	10	hippocampus
**Mitochondrial membrane potential**	4E-F		12	cells		9	cells
**Neurogenesis**	5A-H	6	12	sections	6	12	sections
**Electrophysiology**	6A-E	6-7	13	hippocampus	4	8	hippocampus

Supplementary Table-1. Description of samples. Samples sizes (with sex and genotype) are reported according to experimental procedure.
