# Supplementary material for: Severe hydroxymethylbilane synthase deficiency causes depression-like behavior and mitochondrial dysfunction in a mouse model of homozygous dominant acute intermittent porphyria
Source: Acta Neuropathol Commun. 2020 Mar 20;8:38. doi: 10.1186/s40478-020-00910-z (PMC7082933; doi:10.1186/s40478-020-00910-z)
Supplement: Supplementary file 2 — Additional file 2: Table S2. 228 differentially expressed transcripts were downregulated in HMBS-deficient mice. Columns are as follows: Gene short-name, locus, sample 1+2, value 1+2 (mean reads RPKM), fold change, p-value, q-value. Table S3. 92 differentially expressed transcripts were upregulated in HMBS-deficient mice. Columns are as follows: Gene short-name, locus, sample 1+2, value 1+2 (mean reads RPKM), fold change, p-value, q-value. [file 40478_2020_910_MOESM2_ESM.pdf]

# Supplementary Table-2.

| gene_short_name | locus | sample_1 | sample_2 | value_1 | value_2 | fold change | p_value | q_value |
|---|---|---|---|---|---|---|---|---|
| Gm23490 | chr7:61704518-61829422 | WT | KI | 65,41 | 1,19 | 0,0182 | 5,00E-05 | 0,0042 |
| Pou4f1 | chr14:104461675-104467999 | WT | KI | 0,45 | 0,02 | 0,0426 | 5,00E-05 | 0,0042 |
| 2900076A07Rik,Mir1839 | chr7:81523562-81532521 | WT | KI | 125,19 | 9,03 | 0,0721 | 5,00E-05 | 0,0042 |
| AC154296.1,Gm25791 | chr9:15314841-15314981 | WT | KI | 264,00 | 42,41 | 0,1607 | 5,00E-05 | 0,0042 |
| Gm5741 | chr8:85067567-85067982 | WT | KI | 12,88 | 2,09 | 0,1623 | 5,00E-05 | 0,0042 |
| Mir704,Pdia4 | chr6:47796140-47813430 | WT | KI | 84,82 | 13,90 | 0,1639 | 5,00E-05 | 0,0042 |
| Dvl2,Mir324 | chr11:70000594-70015408 | WT | KI | 45,75 | 8,42 | 0,1841 | 5,00E-05 | 0,0042 |
| Gpr151 | chr18:42578019-42579652 | WT | KI | 3,67 | 0,71 | 0,1930 | 5,00E-05 | 0,0042 |
| Irx2 | chr13:72628819-72634198 | WT | KI | 0,94 | 0,19 | 0,2045 | 5,00E-05 | 0,0042 |
| Gm44783 | chr11:88253367-88256990 | WT | KI | 0,43 | 0,09 | 0,2161 | 5,00E-05 | 0,0042 |
| Six3 | chr17:85600650-85629302 | WT | KI | 2,39 | 0,56 | 0,2353 | 5,00E-05 | 0,0042 |
| Trh | chr6:92242060-92244650 | WT | KI | 1,40 | 0,33 | 0,2391 | 5,00E-05 | 0,0042 |
| Epc1,Mir1893 | chr18:6435950-6516108 | WT | KI | 41,90 | 11,09 | 0,2647 | 5,00E-05 | 0,0042 |
| Chrnb4 | chr9:55028153-55048779 | WT | KI | 0,59 | 0,16 | 0,2764 | 5,00E-05 | 0,0042 |
| Irx1 | chr13:71957920-71963723 | WT | KI | 0,83 | 0,23 | 0,2813 | 5,00E-05 | 0,0042 |
| Lect1 | chr14:79637689-79662170 | WT | KI | 1,75 | 0,54 | 0,3109 | 5,00E-05 | 0,0042 |
| Chrna3 | chr9:55010110-55026562 | WT | KI | 0,88 | 0,30 | 0,3376 | 5,00E-05 | 0,0042 |
| A230065H16Rik | chr12:111406808-111412090 | WT | KI | 2,62 | 0,90 | 0,3452 | 5,00E-05 | 0,0042 |
| Capn11 | chr17:45630203-45659325 | WT | KI | 1,94 | 0,68 | 0,3502 | 5,00E-05 | 0,0042 |
| Snhg15,Snora9 | chr11:6527898-6528746 | WT | KI | 4,18 | 1,59 | 0,3795 | 5,00E-05 | 0,0042 |
| Ctxn3 | chr18:57468515-57478133 | WT | KI | 1,83 | 0,71 | 0,3865 | 5,00E-05 | 0,0042 |
| Lrrc17 | chr5:21483846-21645605 | WT | KI | 2,50 | 0,98 | 0,3934 | 5,00E-05 | 0,0042 |
| Zic4 | chr9:91368969-91389348 | WT | KI | 2,54 | 1,00 | 0,3956 | 5,00E-05 | 0,0042 |
| Crocc2 | chr1:93168724-93231072 | WT | KI | 0,53 | 0,21 | 0,4007 | 5,00E-05 | 0,0042 |
| Gm43398 | chr5:143357337-143403748 | WT | KI | 3,02 | 1,22 | 0,4037 | 5,00E-05 | 0,0042 |
| Wfikkn2 | chr11:94235955-94242707 | WT | KI | 1,14 | 0,47 | 0,4087 | 5,00E-05 | 0,0042 |
| Tcf7l2 | chr19:55741809-55933654 | WT | KI | 13,08 | 5,36 | 0,4097 | 5,00E-05 | 0,0042 |
| Foxp2 | chr6:14901348-15441977 | WT | KI | 1,05 | 0,43 | 0,4146 | 5,00E-05 | 0,0042 |
| Fxyd2 | chr9:45399708-45410278 | WT | KI | 12,88 | 5,39 | 0,4188 | 5,00E-05 | 0,0042 |
| Zic1 | chr9:91358057-91365810 | WT | KI | 14,69 | 6,18 | 0,4203 | 5,00E-05 | 0,0042 |
| Endou | chr15:97711018-97731405 | WT | KI | 0,84 | 0,36 | 0,4328 | 5,00E-05 | 0,0042 |
| Cep131,Gm25948 | chr11:120064429-120086827 | WT | KI | 40,34 | 17,59 | 0,4361 | 5,00E-05 | 0,0042 |
| Syt9 | chr7:107370789-107548656 | WT | KI | 5,34 | 2,34 | 0,4388 | 5,00E-05 | 0,0042 |
| Tac2 | chr10:127724477-127731767 | WT | KI | 9,23 | 4,06 | 0,4399 | 5,00E-05 | 0,0042 |
| Myoc | chr1:162639154-162658219 | WT | KI | 3,72 | 1,66 | 0,4474 | 5,00E-05 | 0,0042 |
| Fam19a4 | chr6:96831202-97060413 | WT | KI | 1,12 | 0,51 | 0,4537 | 5,00E-05 | 0,0042 |
| C3 | chr17:57203969-57228136 | WT | KI | 0,58 | 0,27 | 0,4548 | 5,00E-05 | 0,0042 |
| Fzd10 | chr5:128600843-128604093 | WT | KI | 0,98 | 0,45 | 0,4550 | 5,00E-05 | 0,0042 |
| Slitrk6 | chr14:110748577-110755149 | WT | KI | 1,28 | 0,60 | 0,4661 | 5,00E-05 | 0,0042 |
| Grid2ip | chr5:143357337-143403748 | WT | KI | 2,64 | 1,25 | 0,4726 | 5,00E-05 | 0,0042 |
| Edn1 | chr13:42301269-42307989 | WT | KI | 1,31 | 0,62 | 0,4751 | 5,00E-05 | 0,0042 |
| Apba3,Mir3057 | chr10:81268171-81291267 | WT | KI | 44,87 | 21,40 | 0,4769 | 5,00E-05 | 0,0042 |
| Ramp3 | chr11:6650147-6677475 | WT | KI | 22,06 | 10,56 | 0,4786 | 5,00E-05 | 0,0042 |
| Tmem212 | chr3:27866065-27896368 | WT | KI | 5,10 | 2,45 | 0,4800 | 5,00E-05 | 0,0042 |
| Rec8 | chr14:55618036-55625395 | WT | KI | 1,66 | 0,81 | 0,4890 | 5,00E-05 | 0,0042 |
| Celsr3,Gm23156 | chr9:108826319-108852969 | WT | KI | 174,80 | 87,51 | 0,5006 | 5,00E-05 | 0,0042 |
| Synpo2 | chr3:123076518-123236147 | WT | KI | 6,50 | 3,27 | 0,5027 | 5,00E-05 | 0,0042 |
| Col6a3 | chr1:90765922-90843971 | WT | KI | 1,05 | 0,53 | 0,5061 | 5,00E-05 | 0,0042 |
| Tnnt1 | chr7:4504569-4516004 | WT | KI | 17,66 | 8,96 | 0,5072 | 5,00E-05 | 0,0042 |
| Col24a1 | chr3:145292471-145552011 | WT | KI | 0,56 | 0,28 | 0,5078 | 5,00E-05 | 0,0042 |
| Dnah10 | chr5:124725084-124834308 | WT | KI | 0,43 | 0,22 | 0,5108 | 5,00E-05 | 0,0042 |
| Cox6a2 | chr7:128154018-128206387 | WT | KI | 11,26 | 5,77 | 0,5126 | 5,00E-05 | 0,0042 |
| Ecel1 | chr1:87147654-87156521 | WT | KI | 1,45 | 0,74 | 0,5137 | 5,00E-05 | 0,0042 |
| Hba-a2 | chr11:32296488-32297298 | WT | KI | 41,36 | 21,44 | 0,5184 | 5,00E-05 | 0,0042 |
| Slc17a6 | chr7:51622005-51671119 | WT | KI | 14,36 | 7,48 | 0,5207 | 5,00E-05 | 0,0042 |
| Rab37 | chr11:115091430-115162236 | WT | KI | 6,15 | 3,21 | 0,5226 | 5,00E-05 | 0,0042 |
| Gm9825 | chr6:7982540-7983383 | WT | KI | 13,18 | 6,97 | 0,5287 | 5,00E-05 | 0,0042 |
| Wnt3 | chr11:103774149-103817957 | WT | KI | 2,27 | 1,20 | 0,5302 | 5,00E-05 | 0,0042 |
| Calb2 | chr8:110137501-110168210 | WT | KI | 43,78 | 23,25 | 0,5311 | 5,00E-05 | 0,0042 |
| Nexn | chr3:152236981-152266318 | WT | KI | 2,75 | 1,47 | 0,5351 | 5,00E-05 | 0,0042 |
| Gpr4 | chr7:19212537-19224174 | WT | KI | 2,39 | 1,29 | 0,5377 | 5,00E-05 | 0,0042 |
| Wnt9b | chr11:103727363-103749821 | WT | KI | 0,97 | 0,53 | 0,5427 | 5,00E-05 | 0,0042 |
| Prkcd | chr14:30595355-30626210 | WT | KI | 48,51 | 26,99 | 0,5563 | 5,00E-05 | 0,0042 |
| Ddhd1,Mir5131 | chr14:45587739-45658143 | WT | KI | 34,35 | 19,12 | 0,5566 | 5,00E-05 | 0,0042 |
| Gm15487 | chr6:37806085-37806550 | WT | KI | 216,43 | 120,54 | 0,5570 | 5,00E-05 | 0,0042 |

| | | | | | | | | |
|---|---|---|---|---|---|---|---|---|
| Patj | chr4:98395790-98719603 | WT | KI | 5,76 | 3,23 | 0,5601 | 5,00E-05 | 0,0042 |
| Zfhx3 | chr8:108714643-108961630 | WT | KI | 0,63 | 0,35 | 0,5612 | 5,00E-05 | 0,0042 |
| Frem3 | chr8:80611079-80695356 | WT | KI | 0,63 | 0,35 | 0,5644 | 5,00E-05 | 0,0042 |
| Hcn4 | chr9:58823411-58863175 | WT | KI | 1,51 | 0,85 | 0,5647 | 5,00E-05 | 0,0042 |
| Gm10687 | chr9:44122185-44134485 | WT | KI | 1,06 | 0,60 | 0,5684 | 5,00E-05 | 0,0042 |
| Zic2 | chr14:122475434-122479852 | WT | KI | 9,49 | 5,41 | 0,5703 | 5,00E-05 | 0,0042 |
| Epha8 | chr4:136929418-136956816 | WT | KI | 2,69 | 1,55 | 0,5780 | 5,00E-05 | 0,0042 |
| Syndig1l | chr12:84677277-84698807 | WT | KI | 4,03 | 2,33 | 0,5789 | 5,00E-05 | 0,0042 |
| Mrvi1 | chr7:110868269-110982461 | WT | KI | 1,20 | 0,70 | 0,5790 | 5,00E-05 | 0,0042 |
| Tnc | chr4:63959784-64149924 | WT | KI | 1,61 | 0,93 | 0,5798 | 5,00E-05 | 0,0042 |
| Cdhr3 | chr12:33033795-33092875 | WT | KI | 1,36 | 0,79 | 0,5818 | 5,00E-05 | 0,0042 |
| Lef1 | chr3:131109025-131224356 | WT | KI | 4,76 | 2,79 | 0,5868 | 5,00E-05 | 0,0042 |
| Pdzd3 | chr9:44247306-44251471 | WT | KI | 1,18 | 0,70 | 0,5889 | 5,00E-05 | 0,0042 |
| Prkch | chr12:73584796-73778185 | WT | KI | 1,85 | 1,10 | 0,5964 | 5,00E-05 | 0,0042 |
| Gm27734,Ntan1 | chr16:13819231-13903131 | WT | KI | 35,30 | 21,27 | 0,6025 | 5,00E-05 | 0,0042 |
| Ret | chr6:118151744-118197718 | WT | KI | 2,21 | 1,34 | 0,6057 | 5,00E-05 | 0,0042 |
| Ak7 | chr12:105705981-105782447 | WT | KI | 1,86 | 1,13 | 0,6074 | 5,00E-05 | 0,0042 |
| Rgs16 | chr1:153740348-153749324 | WT | KI | 8,44 | 5,16 | 0,6116 | 5,00E-05 | 0,0042 |
| Krcc1,Mir8112 | chr6:71213939-71322233 | WT | KI | 13,08 | 8,03 | 0,6135 | 5,00E-05 | 0,0042 |
| Plcb4 | chr2:135741829-136014593 | WT | KI | 10,00 | 6,20 | 0,6199 | 5,00E-05 | 0,0042 |
| Plekhg1 | chr10:3740363-3967303 | WT | KI | 11,75 | 7,38 | 0,6282 | 5,00E-05 | 0,0042 |
| Trpc3 | chr3:36620481-36690167 | WT | KI | 3,63 | 2,29 | 0,6310 | 5,00E-05 | 0,0042 |
| Ano1 | chr7:144588548-144738592 | WT | KI | 1,39 | 0,88 | 0,6344 | 5,00E-05 | 0,0042 |
| Vangl1 | chr3:102153582-102206266 | WT | KI | 1,99 | 1,27 | 0,6388 | 5,00E-05 | 0,0042 |
| Igsf5,Pcp4 | chr16:96361667-96525793 | WT | KI | 311,44 | 199,23 | 0,6397 | 5,00E-05 | 0,0042 |
| Rabggtb,Snord45c | chr3:153907288-153913009 | WT | KI | 148,32 | 96,08 | 0,6478 | 5,00E-05 | 0,0042 |
| Cybrd1 | chr2:71117922-71142926 | WT | KI | 1,56 | 1,01 | 0,6490 | 5,00E-05 | 0,0042 |
| Plekhd1 | chr12:80692590-80724214 | WT | KI | 3,02 | 1,97 | 0,6503 | 5,00E-05 | 0,0042 |
| Mir7044,Rasgef1a | chr6:118011437-118091546 | WT | KI | 145,14 | 94,41 | 0,6505 | 5,00E-05 | 0,0042 |
| Gpr153 | chr4:152274231-152285337 | WT | KI | 5,88 | 3,84 | 0,6540 | 5,00E-05 | 0,0042 |
| Col5a3 | chr9:20770049-20815067 | WT | KI | 1,13 | 0,74 | 0,6542 | 5,00E-05 | 0,0042 |
| Cpne9 | chr6:113282306-113305569 | WT | KI | 14,08 | 9,27 | 0,6587 | 5,00E-05 | 0,0042 |
| Foxj1 | chr11:116330703-116335399 | WT | KI | 6,15 | 4,05 | 0,6592 | 5,00E-05 | 0,0042 |
| Amotl1 | chr9:14541965-14615006 | WT | KI | 7,03 | 4,64 | 0,6595 | 5,00E-05 | 0,0042 |
| Cd93 | chr2:148436639-148443563 | WT | KI | 1,75 | 1,16 | 0,6596 | 5,00E-05 | 0,0042 |
| Mir331,Vezt | chr10:93961521-94035817 | WT | KI | 18,55 | 12,24 | 0,6598 | 5,00E-05 | 0,0042 |
| Unc13c | chr9:73479421-73968966 | WT | KI | 3,76 | 2,48 | 0,6602 | 5,00E-05 | 0,0042 |
| Agt | chr8:124556533-124569706 | WT | KI | 20,68 | 13,72 | 0,6636 | 5,00E-05 | 0,0042 |
| Grm4 | chr17:27422386-27513341 | WT | KI | 7,57 | 5,15 | 0,6803 | 5,00E-05 | 0,0042 |
| Gm27322,Smurf2 | chr11:106820065-106920761 | WT | KI | 14,55 | 10,05 | 0,6906 | 5,00E-05 | 0,0042 |
| Gm29562 | chr9:88827521-88828425 | WT | KI | 29,64 | 20,50 | 0,6915 | 5,00E-05 | 0,0042 |
| Vav3 | chr3:109340652-109685698 | WT | KI | 5,21 | 3,60 | 0,6917 | 5,00E-05 | 0,0042 |
| Txnip | chr3:96555787-96566801 | WT | KI | 10,71 | 7,51 | 0,7007 | 5,00E-05 | 0,0042 |
| Rnf152 | chr1:105276913-105361630 | WT | KI | 1,77 | 1,24 | 0,7043 | 5,00E-05 | 0,0042 |
| Cacng5 | chr11:107874604-107915055 | WT | KI | 7,18 | 5,12 | 0,7141 | 5,00E-05 | 0,0042 |
| Rora | chr9:68653785-69388246 | WT | KI | 6,75 | 4,98 | 0,7382 | 5,00E-05 | 0,0042 |
| Slc37a4 | chr9:44396851-44402968 | WT | KI | 15,66 | 11,57 | 0,7389 | 5,00E-05 | 0,0042 |
| Plekhh1 | chr12:79029162-79089670 | WT | KI | 8,57 | 6,42 | 0,7484 | 5,00E-05 | 0,0042 |
| Fxyd6 | chr9:45370184-45396159 | WT | KI | 54,38 | 40,97 | 0,7534 | 5,00E-05 | 0,0042 |
| Chrna4 | chr2:181018379-181039305 | WT | KI | 9,59 | 7,24 | 0,7550 | 5,00E-05 | 0,0042 |
| Mog | chr17:37010742-37023398 | WT | KI | 49,14 | 37,16 | 0,7562 | 5,00E-05 | 0,0042 |
| Ift46 | chr9:44772909-44793447 | WT | KI | 30,44 | 23,02 | 0,7562 | 5,00E-05 | 0,0042 |
| Cit | chr5:115845643-116008947 | WT | KI | 11,60 | 8,84 | 0,7618 | 5,00E-05 | 0,0042 |
| Ttyh2 | chr11:114675430-114720977 | WT | KI | 16,20 | 12,35 | 0,7624 | 5,00E-05 | 0,0042 |
| Cldn11 | chr3:31149919-31164324 | WT | KI | 95,49 | 72,83 | 0,7628 | 5,00E-05 | 0,0042 |
| Ccdc136,Gm26220 | chr6:29396616-29426994 | WT | KI | 103,45 | 79,10 | 0,7646 | 5,00E-05 | 0,0042 |
| Adamts4 | chr1:171250420-171260637 | WT | KI | 9,98 | 7,64 | 0,7648 | 5,00E-05 | 0,0042 |
| Cacng4 | chr11:107732356-107795982 | WT | KI | 10,88 | 8,34 | 0,7664 | 5,00E-05 | 0,0042 |
| Rims3 | chr4:120854818-120896579 | WT | KI | 18,68 | 14,34 | 0,7677 | 5,00E-05 | 0,0042 |
| Camk2d | chr3:126596950-126846326 | WT | KI | 12,13 | 9,31 | 0,7680 | 5,00E-05 | 0,0042 |
| Alas1 | chr9:106233454-106249413 | WT | KI | 41,66 | 32,03 | 0,7688 | 5,00E-05 | 0,0042 |
| Mag | chr7:30899175-30914832 | WT | KI | 117,82 | 90,84 | 0,7710 | 5,00E-05 | 0,0042 |
| Mcam | chr9:44134561-44142727 | WT | KI | 11,35 | 8,79 | 0,7744 | 5,00E-05 | 0,0042 |
| Elmo1 | chr13:20090618-20606528 | WT | KI | 21,33 | 16,54 | 0,7753 | 5,00E-05 | 0,0042 |
| Cnp | chr11:100574903-100581728 | WT | KI | 180,33 | 140,03 | 0,7765 | 5,00E-05 | 0,0042 |
| Myrf | chr19:10208271-10240748 | WT | KI | 19,04 | 14,83 | 0,7793 | 5,00E-05 | 0,0042 |

| | | | | | | | | |
|---|---|---|---|---|---|---|---|---|
| Gm23394,Ralgapa1 | chr12:55602928-55821580 | WT | KI | 33,07 | 25,82 | 0,7806 | 5,00E-05 | 0,0042 |
| Plxnb3 | chrX:73757098-73772514 | WT | KI | 7,76 | 6,07 | 0,7827 | 5,00E-05 | 0,0042 |
| Unc5b | chr10:60762609-60831581 | WT | KI | 9,42 | 7,38 | 0,7830 | 5,00E-05 | 0,0042 |
| Srgap1 | chr10:121780967-122047315 | WT | KI | 4,20 | 3,31 | 0,7862 | 5,00E-05 | 0,0042 |
| Fa2h | chr8:111345140-111393824 | WT | KI | 20,65 | 16,30 | 0,7897 | 5,00E-05 | 0,0042 |
| Mal | chr2:127633225-127656695 | WT | KI | 95,44 | 76,32 | 0,7996 | 5,00E-05 | 0,0042 |
| Gng8 | chr7:16891785-16895435 | WT | KI | 5,09 | 1,85 | 0,3641 | 1,00E-04 | 0,0079 |
| Gpr139 | chr7:119140746-119184603 | WT | KI | 0,41 | 0,22 | 0,5353 | 1,00E-04 | 0,0079 |
| Syt15 | chr14:34220045-34230421 | WT | KI | 0,71 | 0,39 | 0,5515 | 1,00E-04 | 0,0079 |
| Frmpd2 | chr14:33471807-33575269 | WT | KI | 0,56 | 0,31 | 0,5567 | 1,00E-04 | 0,0079 |
| Flnc | chr6:29433275-29461883 | WT | KI | 0,53 | 0,34 | 0,6284 | 1,00E-04 | 0,0079 |
| Mir5113,Tnrc6b | chr15:80711318-80941086 | WT | KI | 10,81 | 7,37 | 0,6816 | 1,00E-04 | 0,0079 |
| Cntn6 | chr6:104492810-104863406 | WT | KI | 2,84 | 2,01 | 0,7066 | 1,00E-04 | 0,0079 |
| Syt6 | chr3:103575230-103646068 | WT | KI | 2,63 | 1,86 | 0,7079 | 1,00E-04 | 0,0079 |
| Plch1 | chr3:63696233-63899472 | WT | KI | 1,95 | 1,39 | 0,7119 | 1,00E-04 | 0,0079 |
| Vim | chr2:13573951-13582826 | WT | KI | 27,56 | 21,61 | 0,7842 | 1,00E-04 | 0,0079 |
| Gm45623 | chr13:56752370-56753827 | WT | KI | 1,37 | 0,64 | 0,4697 | 0,00015 | 0,0110 |
| Slc14a2 | chr18:78146939-78209094 | WT | KI | 0,98 | 0,49 | 0,4981 | 0,00015 | 0,0110 |
| Cfap161 | chr7:83774100-83794880 | WT | KI | 1,40 | 0,73 | 0,5201 | 0,00015 | 0,0110 |
| Zic3 | chrX:58030642-58041736 | WT | KI | 1,53 | 0,87 | 0,5662 | 0,00015 | 0,0110 |
| Gpr88 | chr3:116249653-116253503 | WT | KI | 1,24 | 0,72 | 0,5867 | 0,00015 | 0,0110 |
| Rgs22 | chr15:36009478-36140400 | WT | KI | 0,95 | 0,58 | 0,6064 | 0,00015 | 0,0110 |
| Fam179a | chr17:71673260-71729669 | WT | KI | 1,05 | 0,64 | 0,6136 | 0,00015 | 0,0110 |
| Lrrc55 | chr2:85160777-85196699 | WT | KI | 5,86 | 4,31 | 0,7361 | 0,00015 | 0,0110 |
| Erbb3 | chr10:128567522-128589652 | WT | KI | 3,66 | 2,80 | 0,7648 | 0,00015 | 0,0110 |
| Zfp423 | chr8:87661809-87959595 | WT | KI | 6,21 | 4,78 | 0,7692 | 0,00015 | 0,0110 |
| Sema6a | chr18:47245253-47368868 | WT | KI | 6,96 | 5,36 | 0,7703 | 0,00015 | 0,0110 |
| Pdp1 | chr4:11958183-11966452 | WT | KI | 27,53 | 21,89 | 0,7948 | 0,00015 | 0,0110 |
| Gab1 | chr8:80764437-80881169 | WT | KI | 9,99 | 8,06 | 0,8073 | 0,00015 | 0,0110 |
| Ddr1 | chr17:35681566-35708244 | WT | KI | 35,60 | 28,99 | 0,8144 | 0,00015 | 0,0110 |
| Gsn | chr2:35256379-35307892 | WT | KI | 34,90 | 28,44 | 0,8149 | 0,00015 | 0,0110 |
| AC125167.1,Col3a1 | chr1:45311537-45349706 | WT | KI | 2,17 | 0,67 | 0,3089 | 2,00E-04 | 0,0142 |
| Plp1 | chrX:136822670-136839733 | WT | KI | 752,96 | 554,42 | 0,7363 | 2,00E-04 | 0,0142 |
| Thbs4 | chr13:92751589-92794818 | WT | KI | 5,03 | 3,75 | 0,7442 | 2,00E-04 | 0,0142 |
| Nes | chr3:87971092-87980451 | WT | KI | 2,31 | 1,73 | 0,7471 | 2,00E-04 | 0,0142 |
| Dusp26 | chr8:31089470-31097047 | WT | KI | 20,94 | 16,44 | 0,7853 | 2,00E-04 | 0,0142 |
| Hr | chr14:70553686-70573548 | WT | KI | 5,71 | 4,53 | 0,7932 | 2,00E-04 | 0,0142 |
| Sox10 | chr15:79154912-79164490 | WT | KI | 27,11 | 22,02 | 0,8122 | 2,00E-04 | 0,0142 |
| Mir8115,P2rx7 | chr5:122643910-122691432 | WT | KI | 8,99 | 2,87 | 0,3192 | 0,00025 | 0,0173 |
| Sln | chr9:53850163-53854560 | WT | KI | 0,53 | 0,18 | 0,3473 | 0,00025 | 0,0173 |
| Ebf3 | chr7:137193672-137314445 | WT | KI | 0,64 | 0,32 | 0,4923 | 0,00025 | 0,0173 |
| Lrrc74b | chr16:17544464-17561247 | WT | KI | 1,11 | 0,61 | 0,5498 | 0,00025 | 0,0173 |
| Adgrd1 | chr5:129096749-129204599 | WT | KI | 0,72 | 0,45 | 0,6241 | 0,00025 | 0,0173 |
| Gjc2 | chr11:59175567-59183213 | WT | KI | 11,66 | 8,85 | 0,7586 | 0,00025 | 0,0173 |
| RP23-438P19.12 | chr9:122935811-122942819 | WT | KI | 1,29 | 0,50 | 0,3914 | 3,00E-04 | 0,0204 |
| Adamts19 | chr18:58836763-59053678 | WT | KI | 0,43 | 0,22 | 0,5149 | 3,00E-04 | 0,0204 |
| Zfp521 | chr18:13687012-13972733 | WT | KI | 2,29 | 1,69 | 0,7367 | 3,00E-04 | 0,0204 |
| Gm43305 | chr14:54292442-54297343 | WT | KI | 80,36 | 65,07 | 0,8098 | 3,00E-04 | 0,0204 |
| Sntn | chr14:13670875-13683148 | WT | KI | 0,80 | 0,32 | 0,4040 | 0,00035 | 0,0231 |
| Vipr2 | chr12:116077727-116146261 | WT | KI | 1,24 | 0,78 | 0,6274 | 0,00035 | 0,0231 |
| Mir3062,Pfas | chr11:68985696-69008460 | WT | KI | 18,39 | 13,35 | 0,7256 | 0,00035 | 0,0231 |
| Slc29a1 | chr17:45585199-45599603 | WT | KI | 15,00 | 11,59 | 0,7725 | 0,00035 | 0,0231 |
| Tprn | chr2:25262617-25269885 | WT | KI | 9,87 | 7,73 | 0,7829 | 0,00035 | 0,0231 |
| 2610203C20Rik | chr9:41327259-41615185 | WT | KI | 11,26 | 9,00 | 0,7993 | 0,00035 | 0,0231 |
| Tspan18 | chr2:93201759-93334487 | WT | KI | 9,63 | 7,76 | 0,8060 | 0,00035 | 0,0231 |
| Ugt8a | chr3:125865270-125938619 | WT | KI | 17,95 | 14,57 | 0,8114 | 0,00035 | 0,0231 |
| Tacr1 | chr6:82402474-82560104 | WT | KI | 0,59 | 0,36 | 0,6011 | 4,00E-04 | 0,0258 |
| Pld5 | chr1:175962305-176275298 | WT | KI | 1,30 | 0,80 | 0,6130 | 4,00E-04 | 0,0258 |
| Fhdc1 | chr3:84442197-84480429 | WT | KI | 1,91 | 1,23 | 0,6441 | 4,00E-04 | 0,0258 |
| Vash2 | chr1:190947645-190979296 | WT | KI | 1,42 | 0,95 | 0,6689 | 4,00E-04 | 0,0258 |
| Ntng1 | chr3:109780039-110144011 | WT | KI | 15,98 | 12,35 | 0,7728 | 4,00E-04 | 0,0258 |
| Ccdc170 | chr10:4482501-4562231 | WT | KI | 0,56 | 0,32 | 0,5615 | 0,00045 | 0,0285 |
| Gjc3 | chr5:137953460-137963098 | WT | KI | 15,83 | 12,87 | 0,8131 | 0,00045 | 0,0285 |
| Cxcl5 | chr5:90759377-90761624 | WT | KI | 0,82 | 0,33 | 0,4010 | 5,00E-04 | 0,0311 |
| Zfp474 | chr18:52615914-52639830 | WT | KI | 0,60 | 0,29 | 0,4863 | 5,00E-04 | 0,0311 |
| Odf3b | chr15:89377449-89379254 | WT | KI | 2,96 | 1,78 | 0,6017 | 0,00055 | 0,0335 |

| | | | | | | | | |
|---|---|---|---|---|---|---|---|---|
| Tspan2 | chr3:102720230-102772292 | WT | KI | 51,78 | 38,75 | 0,7483 | 0,00055 | 0,0335 |
| Lpar1 | chr4:58435254-58553628 | WT | KI | 12,12 | 9,69 | 0,7990 | 0,00055 | 0,0335 |
| Nrbp2 | chr15:76085594-76090013 | WT | KI | 75,29 | 62,32 | 0,8278 | 0,00055 | 0,0335 |
| Cntn2 | chr1:132511781-132542939 | WT | KI | 27,47 | 22,86 | 0,8322 | 0,00055 | 0,0335 |
| Msh5 | chr17:35028604-35046726 | WT | KI | 0,23 | 0,45 | 1,9266 | 0,00055 | 0,0335 |
| Stoml3 | chr3:53488726-53508502 | WT | KI | 0,78 | 0,41 | 0,5351 | 6,00E-04 | 0,0363 |
| Krt2 | chr15:101810688-101818169 | WT | KI | 1,82 | 2,59 | 1,4228 | 6,00E-04 | 0,0363 |
| Sema4g | chr19:44989100-45003397 | WT | KI | 9,96 | 8,17 | 0,8206 | 0,00065 | 0,0389 |
| Ptpn4 | chr1:119652466-119837613 | WT | KI | 11,08 | 9,23 | 0,8329 | 0,00065 | 0,0389 |
| Fam216b | chr14:78070716-78090786 | WT | KI | 1,09 | 0,64 | 0,5892 | 7,00E-04 | 0,0407 |
| Nhlh2 | chr3:102010075-102015492 | WT | KI | 1,54 | 1,00 | 0,6503 | 7,00E-04 | 0,0407 |
| Igsf1 | chrX:49782535-49797749 | WT | KI | 5,90 | 4,30 | 0,7295 | 7,00E-04 | 0,0407 |
| Col27a1 | chr4:63215434-63334991 | WT | KI | 2,35 | 1,80 | 0,7684 | 7,00E-04 | 0,0407 |
| Tmem88b | chr4:155781590-155785874 | WT | KI | 26,12 | 21,78 | 0,8339 | 7,00E-04 | 0,0407 |
| 1810041L15Rik | chr15:84379202-84447097 | WT | KI | 18,51 | 15,53 | 0,8393 | 7,00E-04 | 0,0407 |
| Defb1 | chr8:21776598-21795185 | WT | KI | 0,70 | 0,10 | 0,1482 | 0,00075 | 0,0425 |
| Gm44593 | chrX:110634585-110812324 | WT | KI | 0,96 | 0,48 | 0,4949 | 0,00075 | 0,0425 |
| Igfbpl1 | chr4:45809467-45826923 | WT | KI | 1,87 | 1,28 | 0,6832 | 0,00075 | 0,0425 |
| Gdpd5 | chr7:99381413-99461877 | WT | KI | 8,82 | 6,97 | 0,7900 | 0,00075 | 0,0425 |
| Adarb1 | chr10:77290725-77418270 | WT | KI | 43,58 | 36,40 | 0,8353 | 0,00075 | 0,0425 |
| Ccdc40 | chr11:119228571-119265212 | WT | KI | 0,86 | 0,56 | 0,6560 | 8,00E-04 | 0,0447 |
| Hbb-bs | chr7:103826533-103827928 | WT | KI | 242,12 | 194,01 | 0,8013 | 8,00E-04 | 0,0447 |
| Slc16a12 | chr19:34668402-34747289 | WT | KI | 0,45 | 0,71 | 1,5856 | 8,00E-04 | 0,0447 |
| Scube2 | chr7:109798675-109865679 | WT | KI | 0,74 | 0,47 | 0,6345 | 0,00085 | 0,0466 |
| Ppp1r32 | chr19:10474256-10482897 | WT | KI | 1,98 | 1,29 | 0,6487 | 0,00085 | 0,0466 |
| Rgs3 | chr4:62559846-62703018 | WT | KI | 7,15 | 5,51 | 0,7703 | 0,00085 | 0,0466 |
| Aspa | chr11:73304991-73329596 | WT | KI | 13,84 | 10,82 | 0,7820 | 0,00085 | 0,0466 |
| Tmem125 | chr4:118540940-118543728 | WT | KI | 6,04 | 4,45 | 0,7372 | 9,00E-04 | 0,0484 |
| Fmo1 | chr1:162829560-162866610 | WT | KI | 7,69 | 5,71 | 0,7426 | 9,00E-04 | 0,0484 |
| Cacna2d2 | chr9:107399611-107529343 | WT | KI | 7,24 | 5,94 | 0,8203 | 9,00E-04 | 0,0484 |
| Erbin | chr13:103818786-103920514 | WT | KI | 18,34 | 15,32 | 0,8353 | 9,00E-04 | 0,0484 |

**Supplementary Table-2.** 228 differentially expressed transcripts were downregulated in HMBS-deficient mice. Columns are as follows: Gene short-name, locus, sample 1+2, value 1+2 (mean reads RPKM), fold change, p-value, q-value

## Supplementary Table-3.

| gene_short_name | locus | sample_1 | sample_2 | value_1 | value_2 | fold change | p_value | q_value |
|---|---|---|---|---|---|---|---|---|
| Fosl2 | chr5:32135800-32157842 | WT | KI | 7,20 | 8,98 | 1,2478 | 5,00E-05 | 0,0042 |
| Arc | chr15:74669082-74672570 | WT | KI | 63,04 | 79,48 | 1,2609 | 5,00E-05 | 0,0042 |
| Cbl | chr9:44142975-44234049 | WT | KI | 7,80 | 10,25 | 1,3128 | 5,00E-05 | 0,0042 |
| Npy | chr6:49822709-49829507 | WT | KI | 60,01 | 78,79 | 1,3130 | 5,00E-05 | 0,0042 |
| Sst | chr16:23889580-23890844 | WT | KI | 273,95 | 361,66 | 1,3201 | 5,00E-05 | 0,0042 |
| Car12 | chr9:66713685-66766845 | WT | KI | 9,84 | 13,14 | 1,3360 | 5,00E-05 | 0,0042 |
| Slc31a1 | chr4:62360726-62391769 | WT | KI | 5,50 | 7,36 | 1,3385 | 5,00E-05 | 0,0042 |
| Penk | chr4:4133530-4188703 | WT | KI | 15,38 | 20,59 | 1,3388 | 5,00E-05 | 0,0042 |
| Kcnj2 | chr11:111066163-111076821 | WT | KI | 2,10 | 2,85 | 1,3597 | 5,00E-05 | 0,0042 |
| Col9a3 | chr2:180597789-180642708 | WT | KI | 9,08 | 12,60 | 1,3874 | 5,00E-05 | 0,0042 |
| Sc5d | chr9:42251594-42264256 | WT | KI | 14,77 | 20,80 | 1,4081 | 5,00E-05 | 0,0042 |
| Lars2 | chr9:123366926-123462664 | WT | KI | 10,51 | 14,84 | 1,4119 | 5,00E-05 | 0,0042 |
| Ctgf | chr10:24595248-24598683 | WT | KI | 2,66 | 3,96 | 1,4907 | 5,00E-05 | 0,0042 |
| Frem1 | chr4:82897919-83052339 | WT | KI | 0,64 | 0,97 | 1,5306 | 5,00E-05 | 0,0042 |
| Col8a2 | chr4:126286792-126314330 | WT | KI | 1,25 | 1,92 | 1,5396 | 5,00E-05 | 0,0042 |
| Cgnl1 | chr9:71626508-71771602 | WT | KI | 2,33 | 3,76 | 1,6116 | 5,00E-05 | 0,0042 |
| Perp | chr10:18845019-18857073 | WT | KI | 1,31 | 2,18 | 1,6610 | 5,00E-05 | 0,0042 |
| Lamc2 | chr1:153122755-153186447 | WT | KI | 0,35 | 0,59 | 1,6783 | 5,00E-05 | 0,0042 |
| Brd3,Mir7578 | chr2:27445578-27475686 | WT | KI | 12,41 | 20,84 | 1,6790 | 5,00E-05 | 0,0042 |
| Pcolce | chr5:137605102-137611487 | WT | KI | 3,57 | 6,01 | 1,6841 | 5,00E-05 | 0,0042 |
| Drc7 | chr8:95055102-95078141 | WT | KI | 1,64 | 2,77 | 1,6864 | 5,00E-05 | 0,0042 |
| Gm44250 | chr6:5216257-5220486 | WT | KI | 0,73 | 1,24 | 1,7071 | 5,00E-05 | 0,0042 |
| Lepr | chr4:101717403-101815352 | WT | KI | 0,39 | 0,67 | 1,7080 | 5,00E-05 | 0,0042 |
| Ace | chr11:105967944-105989964 | WT | KI | 4,50 | 8,02 | 1,7814 | 5,00E-05 | 0,0042 |
| Slc13a4 | chr6:35267956-35308131 | WT | KI | 2,03 | 3,63 | 1,7865 | 5,00E-05 | 0,0042 |
| Slc37a2 | chr9:37227584-37256149 | WT | KI | 0,62 | 1,13 | 1,8397 | 5,00E-05 | 0,0042 |
| Igfbp6 | chr15:102144361-102149511 | WT | KI | 6,34 | 11,70 | 1,8461 | 5,00E-05 | 0,0042 |
| 1700063D05Rik | chr9:41192445-41217990 | WT | KI | 1,66 | 3,07 | 1,8479 | 5,00E-05 | 0,0042 |
| Enpp2 | chr15:54838897-54920146 | WT | KI | 110,67 | 206,85 | 1,8691 | 5,00E-05 | 0,0042 |
| Igfbp2 | chr1:72824502-72852474 | WT | KI | 22,85 | 42,88 | 1,8769 | 5,00E-05 | 0,0042 |
| Kl | chr5:150952606-150993817 | WT | KI | 7,19 | 13,53 | 1,8822 | 5,00E-05 | 0,0042 |
| Krt18 | chr15:102028215-102032026 | WT | KI | 1,00 | 1,90 | 1,9115 | 5,00E-05 | 0,0042 |
| Gm11549 | chr3:36515056-36521813 | WT | KI | 0,81 | 1,64 | 2,0190 | 5,00E-05 | 0,0042 |
| Fmod | chr1:134037253-134048277 | WT | KI | 0,99 | 2,01 | 2,0215 | 5,00E-05 | 0,0042 |
| RP24-175C20.18 | chr9:44468476-44483803 | WT | KI | 3,65 | 7,55 | 2,0701 | 5,00E-05 | 0,0042 |
| Trpv4 | chr5:114622151-114658421 | WT | KI | 1,06 | 2,21 | 2,0805 | 5,00E-05 | 0,0042 |
| Sostdc1 | chr12:36314168-36318452 | WT | KI | 3,14 | 6,57 | 2,0903 | 5,00E-05 | 0,0042 |
| Igf2 | chr7:142650765-142670356 | WT | KI | 12,35 | 26,56 | 2,1494 | 5,00E-05 | 0,0042 |
| Mir6904,Parp1 | chr1:180568928-180601254 | WT | KI | 1069,91 | 2304,32 | 2,1538 | 5,00E-05 | 0,0042 |
| 77,Mir382,Mir410,Mir412, | chr12:109729763-109749460 | WT | KI | 16,03 | 35,08 | 2,1881 | 5,00E-05 | 0,0042 |
| Akt1s1,Mir707 | chr7:44812255-44855421 | WT | KI | 43,52 | 95,53 | 2,1951 | 5,00E-05 | 0,0042 |
| Sulf1 | chr1:12692276-12861192 | WT | KI | 1,45 | 3,20 | 2,2055 | 5,00E-05 | 0,0042 |
| Cldn1 | chr16:26356641-26371841 | WT | KI | 0,58 | 1,31 | 2,2399 | 5,00E-05 | 0,0042 |
| Kctd12 | chr14:102976580-102982637 | WT | KI | 10,35 | 23,30 | 2,2516 | 5,00E-05 | 0,0042 |
| Abca4 | chr3:122044442-122227370 | WT | KI | 0,75 | 1,71 | 2,2858 | 5,00E-05 | 0,0042 |
| Wdr86 | chr5:24711737-24730727 | WT | KI | 0,51 | 1,18 | 2,3075 | 5,00E-05 | 0,0042 |
| Clic6 | chr16:92498133-92541243 | WT | KI | 2,32 | 5,65 | 2,4309 | 5,00E-05 | 0,0042 |
| Agxt2,Gm21973,Prlr | chr15:10177654-10410153 | WT | KI | 0,62 | 1,60 | 2,5859 | 5,00E-05 | 0,0042 |
| 1500015O10Rik | chr1:43730601-43743895 | WT | KI | 7,29 | 18,91 | 2,5928 | 5,00E-05 | 0,0042 |
| Slc4a5 | chr6:83219827-83304945 | WT | KI | 0,76 | 2,13 | 2,8095 | 5,00E-05 | 0,0042 |
| Col8a1 | chr16:57624257-57754737 | WT | KI | 0,44 | 1,26 | 2,8524 | 5,00E-05 | 0,0042 |
| Folr1 | chr7:101858330-101870788 | WT | KI | 3,40 | 10,23 | 3,0127 | 5,00E-05 | 0,0042 |
| F5 | chr1:164115263-164220277 | WT | KI | 0,69 | 2,10 | 3,0224 | 5,00E-05 | 0,0042 |
| Kcne2 | chr16:92292388-92298129 | WT | KI | 1,35 | 4,10 | 3,0429 | 5,00E-05 | 0,0042 |
| Aqp1 | chr6:55336431-55348555 | WT | KI | 1,08 | 3,41 | 3,1481 | 5,00E-05 | 0,0042 |
| Klhdc7a | chr4:139960219-139968026 | WT | KI | 2,26 | 7,54 | 3,3349 | 5,00E-05 | 0,0042 |
| Prr32 | chrX:45090903-45092791 | WT | KI | 0,69 | 2,33 | 3,3787 | 5,00E-05 | 0,0042 |
| Cldn2 | chrX:139800827-139811386 | WT | KI | 0,63 | 2,36 | 3,7288 | 5,00E-05 | 0,0042 |
| Steap1 | chr5:5736316-5749326 | WT | KI | 0,43 | 1,61 | 3,7652 | 5,00E-05 | 0,0042 |
| Ttr | chr18:20558073-20674324 | WT | KI | 318,33 | 1317,99 | 4,1403 | 5,00E-05 | 0,0042 |
| Mir1907,Trps1 | chr15:50654751-50890041 | WT | KI | 4,80 | 24,07 | 5,0101 | 5,00E-05 | 0,0042 |
| Gm26325,Rnf225 | chr7:12927415-12931072 | WT | KI | 0,39 | 2,93 | 7,5852 | 5,00E-05 | 0,0042 |
| Glce,Mir5133 | chr9:62057247-62122610 | WT | KI | 6,98 | 73,73 | 10,5609 | 5,00E-05 | 0,0042 |
| Gm22137,Gm7541 | chr16:8623162-8623720 | WT | KI | 0,27 | 8,43 | 31,5050 | 5,00E-05 | 0,0042 |
| Gm29718 | chr1:136793033-136793510 | WT | KI | 0,77 | 0,00 | #NAME? | 5,00E-05 | 0,0042 |

| | | | | | | | | |
|---|---|---|---|---|---|---|---|---|
| Mir1946b | chr9:21613443-21613576 | WT | KI | 9,82 | 0,00 | #NAME? | 5,00E-05 | 0,0042 |
| Junb | chr8:84976909-84978718 | WT | KI | 33,73 | 42,64 | 1,2641 | 1,00E-04 | 0,0079 |
| Cab39l | chr14:59440980-59548903 | WT | KI | 8,84 | 11,37 | 1,2862 | 1,00E-04 | 0,0079 |
| Ldlr | chr9:21723482-21749919 | WT | KI | 4,84 | 6,27 | 1,2972 | 1,00E-04 | 0,0079 |
| Spint2 | chr7:29256340-29281912 | WT | KI | 12,78 | 17,76 | 1,3898 | 1,00E-04 | 0,0079 |
| Pttg1 | chr11:43420249-43426251 | WT | KI | 8,74 | 13,69 | 1,5663 | 1,00E-04 | 0,0079 |
| Bglap3 | chr3:88368615-88372743 | WT | KI | 0,28 | 0,71 | 2,5381 | 1,00E-04 | 0,0079 |
| Vip | chr10:5639217-5647617 | WT | KI | 10,01 | 13,37 | 1,3353 | 0,00015 | 0,0110 |
| AY036118 | chr17:39846957-39848827 | WT | KI | 135,46 | 168,87 | 1,2467 | 0,00025 | 0,0173 |
| Fap | chr2:62500942-62574075 | WT | KI | 0,25 | 0,51 | 2,0595 | 4,00E-04 | 0,0258 |
| Gm27762,St7 | chr6:17693936-17943025 | WT | KI | 12,78 | 16,47 | 1,2887 | 0,00045 | 0,0285 |
| RP24-329C11.8,Rnf26 | chr9:44066326-44121960 | WT | KI | 3,64 | 7,52 | 2,0639 | 0,00045 | 0,0285 |
| Pla2g5 | chr4:138799243-138863482 | WT | KI | 0,52 | 1,23 | 2,3710 | 0,00045 | 0,0285 |
| Col5a1 | chr2:27882924-28039514 | WT | KI | 1,68 | 2,18 | 1,3013 | 5,00E-04 | 0,0311 |
| Crhbp | chr13:95431370-95444831 | WT | KI | 8,74 | 11,44 | 1,3090 | 5,00E-04 | 0,0311 |
| Nlrx1 | chr9:44252716-44268599 | WT | KI | 1,70 | 2,48 | 1,4581 | 5,00E-04 | 0,0311 |
| Crhr1 | chr11:104132854-104175523 | WT | KI | 4,26 | 5,71 | 1,3386 | 0,00065 | 0,0389 |
| Rprm | chr2:54084092-54085552 | WT | KI | 16,98 | 21,58 | 1,2711 | 7,00E-04 | 0,0407 |
| Cd27,Mir8113 | chr6:125215550-125241249 | WT | KI | 0,07 | 0,99 | 14,4083 | 7,00E-04 | 0,0407 |
| Satb2 | chr1:56793980-57050084 | WT | KI | 2,45 | 3,25 | 1,3244 | 0,00075 | 0,0425 |
| Pon3 | chr6:5220851-5256286 | WT | KI | 0,84 | 1,37 | 1,6211 | 0,00075 | 0,0425 |
| RP24-175C20.19 | chr9:44468476-44483803 | WT | KI | 1,63 | 2,94 | 1,7976 | 0,00075 | 0,0425 |
| Fam180a | chr6:35312667-35326141 | WT | KI | 0,19 | 0,42 | 2,2665 | 8,00E-04 | 0,0447 |
| Kcnh3 | chr15:99224975-99242817 | WT | KI | 27,25 | 32,61 | 1,1968 | 0,00085 | 0,0466 |
| Cckbr | chr7:105425730-105470898 | WT | KI | 3,20 | 4,32 | 1,3494 | 0,00085 | 0,0466 |
| Lamp5 | chr2:136057926-136069917 | WT | KI | 12,32 | 15,28 | 1,2400 | 9,00E-04 | 0,0484 |
| Fzd4 | chr7:89404354-89413134 | WT | KI | 2,14 | 2,74 | 1,2847 | 9,00E-04 | 0,0484 |

**Supplementary Table-3.** 92 differentially expressed transcripts were upregulated in HMBS-deficient mice. Columns are as follows: Gene short-name, locus, sample 1+2, value 1+2 (mean reads RPKM), fold change, p-value, q-value
