# Supplementary material for: Severe hydroxymethylbilane synthase deficiency causes depression-like behavior and mitochondrial dysfunction in a mouse model of homozygous dominant acute intermittent porphyria
Source: Acta Neuropathol Commun. 2020 Mar 20;8:38. doi: 10.1186/s40478-020-00910-z (PMC7082933; doi:10.1186/s40478-020-00910-z)
Supplement: Supplementary file 3 — Additional file 3: Figure S1. Basal TMRM fluorescence in cultured hippocampal neurons and pharmacological intervention with ATP synthase inhibitor (Oligomycin), complex III inhibitor (Antimycin A) and protonophore FCCP in KI and WT mice; n = 5–9 cells per genotype. TMRM, tetramethyl rhodamine ethyl ester, FCCP, carbonyl cyanide-4-(trifluoromethoxy)phenylhydrazone. [file 40478_2020_910_MOESM3_ESM.pdf]

Supplementary Figure-1.

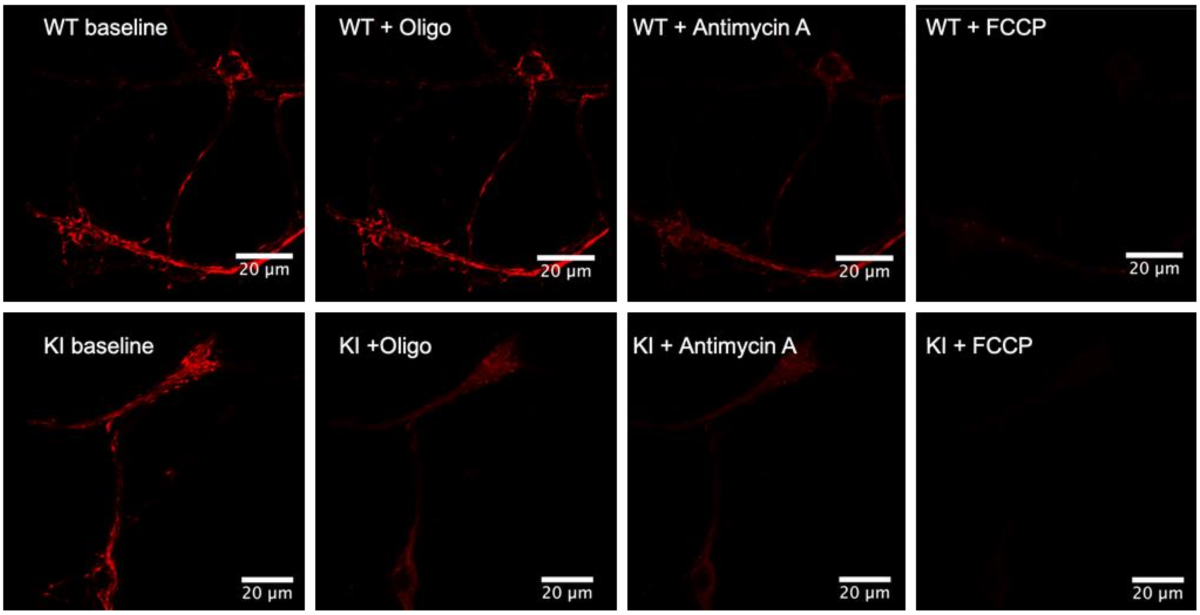

Supplementary Figure-1. Basal TMRM fluorescence in cultured hippocampal neurons and pharmacological intervention with ATP synthase inhibitor (Oligomycin), complex III inhibitor (Antimycin A) and protonophore FCCP in KI and WT mice; n=5-9 cells per genotype. TMRM, tetramethyl rhodamine ethyl ester, FCCP, carbonyl cyanide-4-(trifluoromethoxy)phenylhydrazone
