# Supplementary material for: Severe hydroxymethylbilane synthase deficiency causes depression-like behavior and mitochondrial dysfunction in a mouse model of homozygous dominant acute intermittent porphyria
Source: Acta Neuropathol Commun. 2020 Mar 20;8:38. doi: 10.1186/s40478-020-00910-z (PMC7082933; doi:10.1186/s40478-020-00910-z)
Supplement: Supplementary file 4 — Additional file 4: Figure S2. Neuropathohistological evaluation of hippocampal coronal sections stained with hematoxylin-eosin (H&E), SMI-31 (phosphorylated epitope in neurofilament) and SMI-32 (non-phosphorylated epitope in neurofilament) of HMBS-deficient (KI) and wildtype (WT) mice; n = 8–12 per genotype. [file 40478_2020_910_MOESM4_ESM.pdf]

**Supplementary Figure-2.**

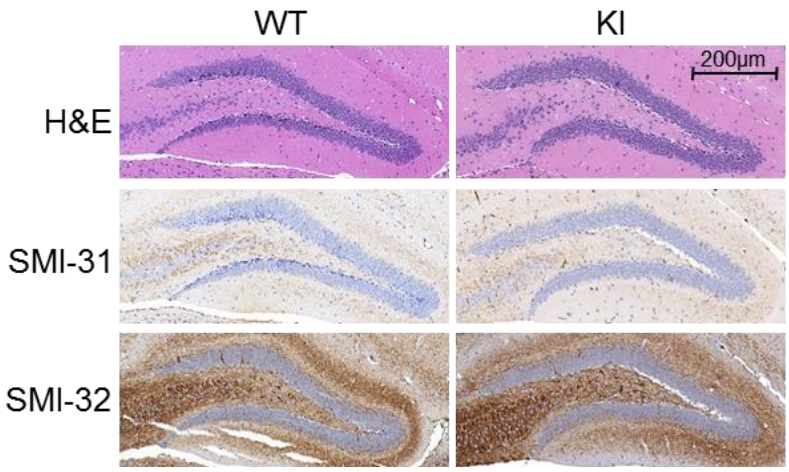


**Supplementary Figure-2.** Neuropathohistological evaluation of hippocampal coronal sections stained with hematoxylin-eosin (H&E), SMI-31 (phosphorylated epitope in neurofilament) and SMI-32 (non-phosphorylated epitope in neurofilament) of HMBS-deficient (KI) and wildtype (WT) mice; n=8-12 per genotype.
